# In Vitro vs. In Vivo Transcriptomic Approach Revealed Core Pathways of Nitrogen Deficiency Response in Tea Plant (*Camellia sinensis* (L.) Kuntze)

**DOI:** 10.3390/ijms252111726

**Published:** 2024-10-31

**Authors:** Lidiia Samarina, Lyudmila Malyukova, Songbo Wang, Aleksandr Bobrovskikh, Alexey Doroshkov, Ruset Shkhalakhova, Karina Manakhova, Natalia Koninskaya, Alexandra Matskiv, Alexey Ryndin, Elena Khlestkina, Yuriy Orlov

**Affiliations:** 1 Federal Research Centre, The Subtropical Scientific Centre of the Russian Academy of Sciences, 354002 Sochi, Russia; malukovals@mail.ru (L.M.); wangsongbo@bio-intelligence.cn (S.W.); shhalahova1995@mail.ru (R.S.); karina.khusniyarova@gmail.com (K.M.); natakoninskaya@mail.ru (N.K.); matskiv_a@mail.ru (A.M.); subplod@mail.ru (A.R.); 2 Center of Genetics and Life Sciences, Sirius University of Science and Technology, 354340 Sirius, Russia; director@vir.nw.ru; 3 The Federal Research Center Institute of Cytology and Genetics, Siberian Branch of the Russian Academy of Sciences, 630090 Novosibirsk, Russia; avb@bionet.nsc.ru (A.B.); ad@bionet.nsc.ru (A.D.); 4 Federal Research Center N. I. Vavilov All-Russian Institute of Plant Genetic Resources (VIR), 190000 Saint Petersburg, Russia; 5 Agrarian and Technological Institute, Peoples’ Friendship University of Russia, 117198 Moscow, Russia

**Keywords:** caffeine, catechins, cell wall, cytochromes, L-theanine, nitrogen uptake, phenylpropanoid pathway

## Abstract

For the first time, we used an *in vitro* vs. *in vivo* experimental design to reveal core pathways under nitrogen deficiency (ND) in an evergreen tree crop. These pathways were related to lignin biosynthesis, cell redox homeostasis, the defense response to fungus, the response to Karrikin, amino acid transmembrane transport, the extracellular region, the cellular protein catabolic process, and aspartic-type endopeptidase activity. In addition, the mitogen-activated protein kinase pathway and ATP synthase (ATP)-binding cassette transporters were significantly upregulated under nitrogen deficiency in vitro and in vivo. Most of the MAPK downstream genes were related to calcium signaling (818 genes) rather than hormone signaling (157 genes). Moreover, the hormone signaling pathway predominantly contained auxin- and abscisic acid-related genes, indicating the crucial role of these hormones in ND response. Overall, 45 transcription factors were upregulated in both experiments, 5 *WRKYs*, 3 *NACs*, 2 *MYBs*, 2 *ERFs*, *HD-Zip*, *RLP12*, *bHLH25*, *RADIALIS*-like, and others, suggesting their ND regulation is independent from the presence of a root system. Gene network reconstruction displayed that these transcription factors participate in response to fungus/chitin, suggesting that nitrogen response and pathogen response have common regulation. The upregulation of lignin biosynthesis genes, cytochrome genes, and strigalactone response genes was much more pronounced under *in vitro* ND as compared to *in vivo* ND. Several cell wall-related genes were closely associated with cytochromes, indicating their important role in flavanols biosynthesis in tea plant. These results clarify the signaling mechanisms and regulation of the response to nitrogen deficiency in evergreen tree crops.

## 1. Introduction

Nitrogen (N) is an essential element, being a part of proteins, nucleic acids, enzymes, hormones, and bioactive compounds. Under conditions of insufficient N supply, the synthesis of enzymes slows down, leading to the disruption of metabolism and yield decrease [1]. Nitrogen deficiency is one of the main limiting factors for production of many crops [1,2]. It is well known that plants uptake nitrogen from the soil in the form of nitrates, ammonium, urea, and amino acids, and different plant genotypes can assimilate nitrogen with different efficiencies. Under low-nitrogen conditions, some varieties may be characterized by better agricultural quality than the others [3]. Understanding molecular mechanisms of ND-response and the identification of plant genotypes with a high efficiency of nitrogen utilization are important for sustainable agriculture.

The mechanisms of nitrogen assimilation and utilization are complex, especially in perennial tree crops. By the way, for the last 60 years, the global use of inorganic N fertilizers has been increased 10 times, while it was estimated that only 6–50% of fertilizers are taken up by plants. The efficiency of fertilizer application is dependent on the fertilizer, plant genotype and soil type, climatic conditions, and agricultural practices [1,4]. Nitrogen dosages are especially problematic in tree crops due to the slow distribution and remobilization through plant tissues [5]. This can lead to an excessive application of nitrogen fertilizers with following soil acidification, disruption of the soil microbiome, and environmental damage [6,7,8]. Thus, understanding the mechanisms of nitrogen use efficiency can help breeders to obtain better yield quality under low-nitrogen conditions [3,4,9].

Tea plant (*Camellia sinensis* (L.) Kuntze) is an important evergreen tree crop widely grown in more than 52 countries [10,11]. Recent progress on tea plant genomics allows us to consider it as one of the model perennial evergreen tree crops for gene mining and unraveling molecular stress responses, particularly the response to ND [12,13]. N application affects the quality of tea plants by regulation of the accumulation of flavanols, amino acids, and methylxanthines [5,14,15,16,17]. Recently, a set of genes were identified which are involved in nitrogen transport and metabolism (*AMT*, *AQP*, *NRT*, *GOGAT*, and *GS*), N uptake regulation (*AMT1.2*, *NRT2.4*, and *PIP*), N assimilation (*GS* and *GDH*), and root amino acid transport (*LHT*) [3,15,18,19,20,21,22,23]. However, the molecular connection of flavanol biosynthesis and nitrogen-responsive genes has not been sufficiently studied, while the knowledge of signaling and regulatory mechanisms is still fragmentary. Moreover, most of the published studies reported the transcriptional response of roots, while leaf response needs further investigation because it can help to understand better the molecular connection of tea leaf quality and ND response.

In addition, all published data were obtained in potted or field experiments. Meanwhile, an *in vitro* vs. *in vivo* approach can provide a new understanding of the core fundamental mechanisms of ND responses. Few studies used an *in vitro* approach to evaluate the effect of abiotic stress on tree crops. For example, the effect of mineral salts on growth and secondary metabolism was studied in apple callus [24], grape callus [25]), and carrot callus [26]. However, these are mostly metabolomic studies, and the effect of mineral salts on the transcription response of shoots was not evaluated. Thus, the goal of our research was to reveal common molecular mechanisms and regulatory networks in leaf tissues under nitrogen deficiency *in vitro* and *in vivo*. Comparative HPLC RNAseq and gene network reconstruction approaches provide new knowledge on cytochrome-related DEGs and the signaling and regulatory mechanisms of ND response in evergreen tree crops.

## 2. Results

### 2.1. Leaf Quality Evaluation under ND In Vitro and In Vivo

**Figure 1:**
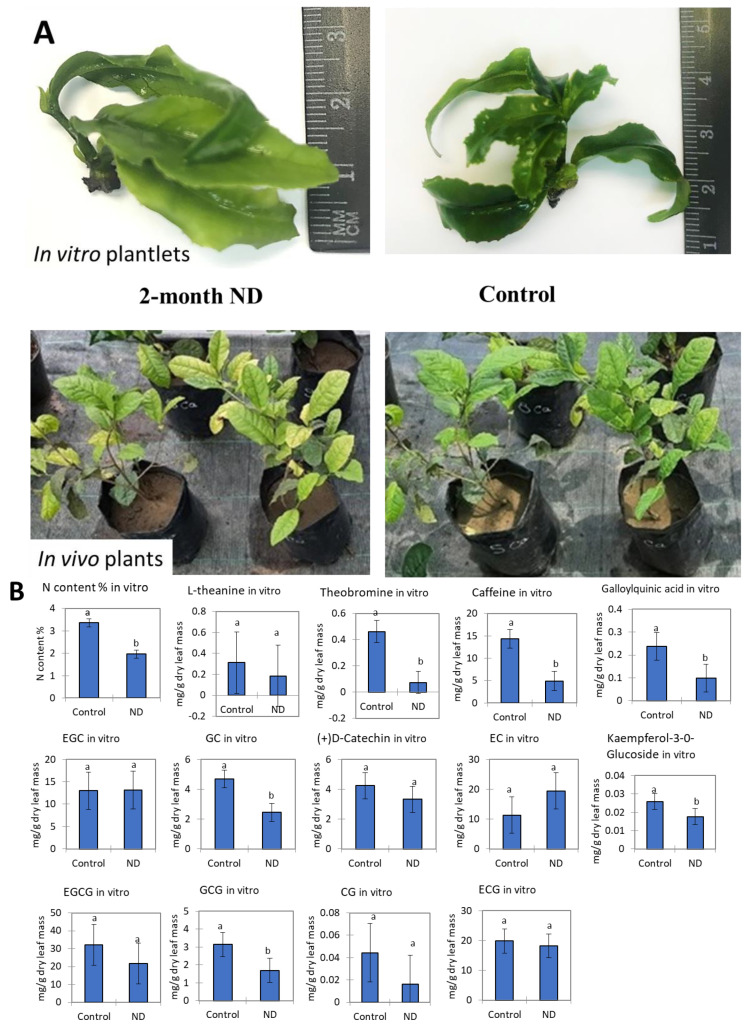
(**A**)—experimental tea plants of cv. Kolkhida *in vitro* and *in vivo*. (**B**)—effect of nitrogen deficiency on L-theanine, methylxanthines, and flavanols (mg g^−1^ dry leaf mass) *in vitro*. Different lowercase letters indicate significant differences according to Tukey’s test at *p* ≤ 0.05. Raw data, *in vivo* data, and detailed statistics can be found in the Supplementary File S1.

*In vitro* ND resulted in a two-fold decrease in leaf N content in tea plantlets. In addition, a significant decrease in methylxanthines, galloylquinic acid, kaempferol-3-O-glucoside, and two catechins, namely GC and GCG, was observed. Particularly, theobromine content was decreased about 10-fold, caffeine content about three-fold, galloylquinic acid, GC, and GCG about two-fold. No effect of ND was observed on EGC, EGCG, catechin, CG, EC, and ECG (Figure 1).

*In vivo* ND resulted in a significant decrease in leaf N content by about 1.5–2.0-fold. Additionally, L-theanine and caffeine contents were decreased by 5-fold. However, a significant increase in most catechins was observed under 2 and 4 months of ND, namely EC, GC, catechin, and EGC. In particular, the content of EC was twice greater, GC three times greater, catechin six times greater, and EGC 1.5 times greater under ND as compared to the control (Supplementary Figure S1). The content of gallate catechins ECG, GCG, and EGCG was also significantly increased after 2 months of ND, but decreased or has not been changed after 4 months of ND *in vivo*.

### 2.2. GO and KEGG Pathways Enrichment under ND In Vitro and In Vivo

*In vitro* ND resulted in 14 significantly enriched KEGG pathways (Photosynthesis—antenna proteins (0 up/9 downregulated DEGs), Phenylpropanoid biosynthesis (19 up/22 down), Taurine and hypotaurine metabolism (4 up/1 down), ABC transporters (18 up/4 down), Flavonoid biosynthesis (4 up/16 down), Stilbenoid, diarylheptanoid and gingerol biosynthesis (1 up/13 down), Starch and sucrose metabolism (30 up/8 down), Galactose metabolism (17 up/2 down), Glutathione metabolism (8 up/8 down), MAPK signaling pathway—plant (27 up/3down), Cutin, suberine, and wax biosynthesis (5 up/3 down), Amino sugar and nucleotide sugar metabolism (17 up/5 down), Arachidonic acid metabolism (6 up/1 down), and Brassinosteroid biosynthesis (6 up/1 down)) (Supplementary File S2).

*In vivo* ND resulted in eight significantly enriched KEGG pathways: Flavonoid biosynthesis, Phenylpropanoid biosynthesis, Starch and sucrose metabolism, Carbon fixation, Nitrogen metabolism, Photosynthesis, Cyanoamino acid metabolism, and Brassinosteroid biosynthesis (Supplementary File S3).

**Figure 2:**
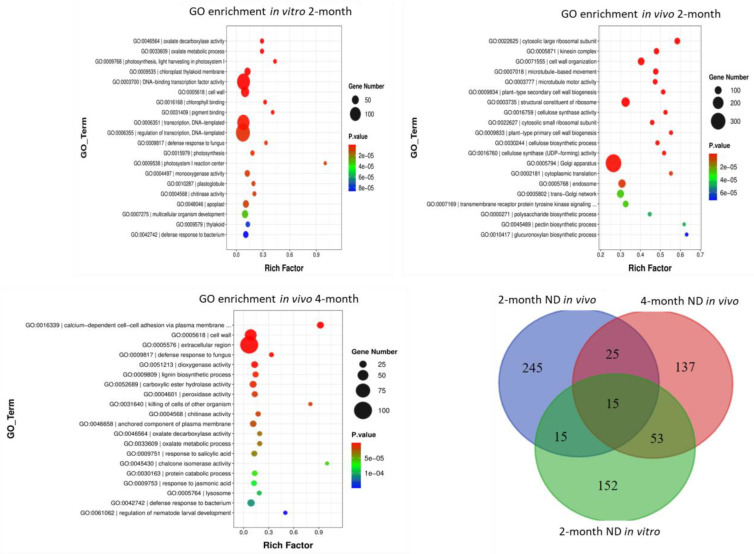
Top GO terms significantly enriched under nitrogen deficiency in tea plants *in vitro*, and *in vivo*. Venn diagram displays common and specific GO terms for all experiments.

*In vitro* GO analysis displayed in total 272 upregulated and 137 downregulated DEGs of biological processes with a Log *p* value ≥ 4. The highest number of DEGs were related to multicellular organism development, DNA-templated transcription, and regulation of DNA-templated transcription (Figure 2). GO terms of cellular components included 163 upregulated and 161 downregulated DEGs and the highest number of DEGs with a Log *p* value ≥ 4 were related to the following terms: thylakoid (GO: 0009579), chloroplast thylakoid membrane (GO: 0009535), plastoglobule (GO: 0010287), photosystem I reaction center (GO: 0009538), cell wall (GO: 0005618), apoplast (GO: 0048046), and extracellular region (GO: 0005576). GO terms of molecular function included 193 upregulated and 83 downregulated DEGs and the highest number of DEGs with a Log *p* value ≥ 4 were related to the following terms: photosynthesis, light harvesting in photosystem I (GO: 0016168), peroxidase activity (GO: 0004601), monooxygenase activity (GO: 0004497), copper ion binding (GO: 0005507), transcription regulatory region DNA binding (GO: 0044212), and DNA binding transcription factor activity (GO: 0003700).

*In vivo* GO analysis showed downregulation of most DEGs related to cell wall organization, such as cell wall biogenesis (GO:0009833 and GO:0009834), cellulose biosynthesis (GO:0030244), the pectin biosynthetic process (GO:0045489), and microtubule-based movement (GO:0007018), with a log_10_ (*p* value) of about 3–11. Among the GO terms of cellular components, the downregulation of most DEGs related to kinesin complex (GO:0005871), microtubule (GO:0005874), endosome (GO:0005768), trans-Golgi network (GO:0005802), Golgi apparatus (GO:0005794), and plasma membrane (GO:0005886) was observed. However, the upregulation of most DEGs related to cytosolic large ribosomal subunit (GO:0022625), cytosolic small ribosomal subunit (GO:0022627), and DNA-directed RNA polymerase II core complex (GO:0005665) was detected. Among the molecular function group, the downregulation of most DEGs related to cellulose synthase activity (GO:0016759, GO:0016760), ATP-dependent microtubule motor activity (GO:0003777, GO:0008574), transferase activity, and transferring glycosyl groups (GO:0016757) was observed. However, the DEGs related to transition metal ion binding (GO:0046914), structural constituent of ribosome (GO:0003735), and RNA polymerase III activity (GO:0001056) were mostly upregulated (Figure 2, Supplementary File S3).

The Venn diagram displays 15 common GO terms for in vitro and in vivo experiments. These terms are response to Karrikin (GO:0080167), —transition metal ion binding and homeostasis (GO:0046914; GO:0046916), cell redox homeostasis (GO:0045454), —amino acid transmembrane transport (GO:0003333), protein catabolic process (GO:0030163, GO:0051603), cell wall macromolecule catabolic process (GO:0016998), extracellular region (GO:0005576), —cell wall and lignin biosynthesis (GO:0005618, GO:0009809), defense response to fungus and to chitin (GO:0050832, GO:0010200), —aspartic-type endopeptidase activity (GO:0004190), and lysosome (GO:0005764). These pathways are consistently associated with transcriptome changes in response to nitrogen deficiency.

### 2.3. Key Common DEGs Significantly Affected by ND In Vitro and In Vivo

**Figure 3:**
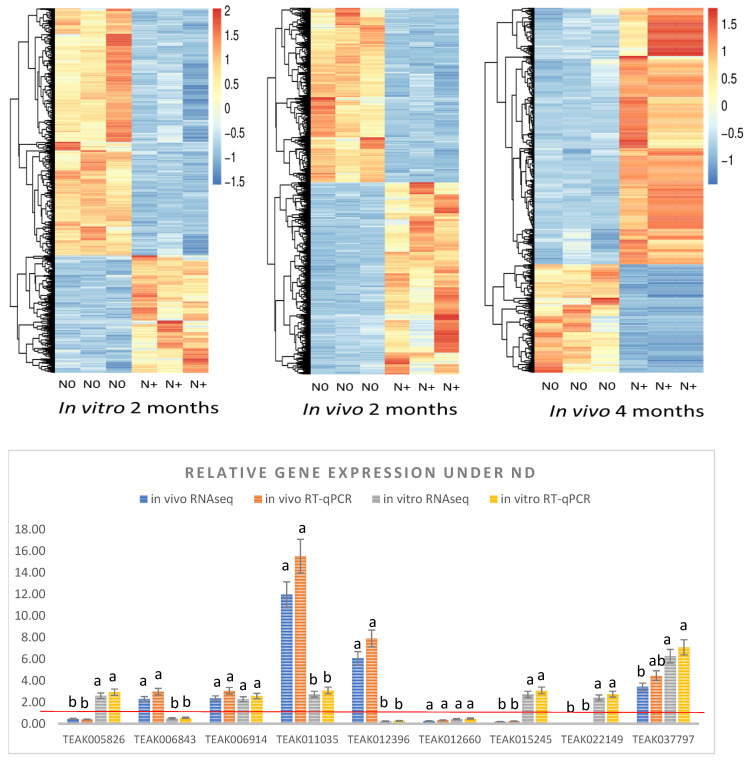
Heat maps of all DEGs and barplot of RT-qPCR validation of gene expression. Bars represent standard errors. Red line indicates the relative gene expression in control treatment (N+). TEAK005826 (*PAL*), TEAK006843 (*F 3',5'-H*), TEAK006914 (*CYP450 82C4*), TEAK011035 (*F 3'-H*), TEAK012396 (*Cytochrome P450 78A5*), TEAK012660 (*F 3',5'-H*), TEAK015245 (*PAL*), TEAK022149 (*CYP450 82A3*), and TEAK037797 (*CYP736A54*). Different small letters indicate significant difference at *p* < 0.05 according to Tukey’s range test with multiple comparisons model.

In total, 1733 (2-month ND in vitro), 7089 (2-month ND *in vivo*), and 1323 (4-month ND *in vivo*) significant DEGs were identified (Figure 3, Supplementary File S4). The results of RNAseq were generally consistent with the results of RT-qPCR. Interestingly, two PAL genes were upregulated *in vitro* but downregulated *in vivo* after 2 months of ND. Also, TEAK006843 (*F 3',5'-H*) and TEAK011035 (*F 3'-H*) were downregulated *in vitro*, but upregulated *in vivo*; however, TEAK012660 (*F 3',5'-H*) was downregulated in both experiments. Thus, according to RT-qPCR and RNAseq, flavanol biosynthesis DEGs were differentially regulated, which is consistent with HPLC results on flavanol accumulation.

**Figure 4:**
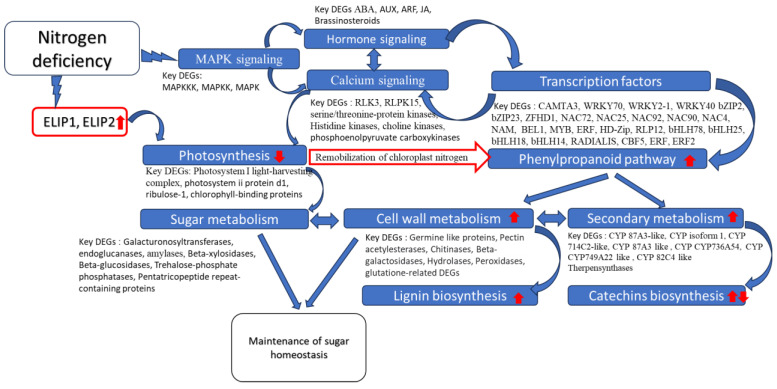
The general scheme of leaf ND response in tea plant *in vitro* and *in vivo*. Small red arrows display up/downregulation of the respective process. More details can be found in Supplementary File S4.

Based on the analysis of significant DEGs, the general scheme of the core ND response is proposed (Figure 4). In this scheme, the signaling and regulation of the key responsive pathways were clarified, particularly the following:

(A) Calcium signaling included 818 DEGs with
  - 40 DEGs directly related to MAPK signaling: *Serine-threonine kinases, protein-tyrosine-phosphatases, histidine kinases, leucine-rich repeat receptors, and sucrose non-fermenting-1-related protein kinases.*
  - 28 DEGs of *calcium-dependent protein kinases, calcium/calmodulin receptors, and CDPK-related kinases.*
  - 39 DEGs of hormonal signaling: *histidine kinases, leucine-rich repeat receptor-like protein kinases, LRR receptor-like serine/threonine protein kinases, Serine/threonine protein kinases, and sucrose non-fermenting-1-related protein kinases.*
(B) Hormone signaling included 157 DEGs with
  - 24 DEGs directly related to MAPK signaling: *abscisic acid receptors*, *Dentin sialoproteins*, *EIN2-CEND*, *ethylene-responsive TFs*, *CUMW_237540*, *inducer of CBF expression 2*, *pathogenesis-related protein 1*, LIGHT-DEPENDENT SHORT HYPOCOTYLS 10, *bHLH14*, *bHLH18*, *bHLH25*, *protein phosphatase 2C 24*, and *protein phosphatase 2C 16*,
  - 44 DEGs of auxin signaling: *auxin early response proteins GH3.1*, *GH3.9*, *SAUR23*, *SAUR41*, and *SAUR68*, *auxin responsive factors ARF2*, *ARF5*, and *ARF6*, *auxin transporter proteins*, *auxin-induced proteins*, *E3 ubiquitin proteins*, *EIN2-CEND*, *GH3 auxin-responsive promoters*, *indole-3-acetic acid-amido synthetase GH3.1*, and *transport inhibitor response 1*.
  - 34 DEGs of ABA signaling: *abscisic acid receptors, ACT domain-containing proteins, bZIP1, bZIP8, Hb-ZIP, WCOR413, MFT1, general transcriptional corepressor trfA, bHLH14, MYB-related TF, DMP2, DMP6, IQ-DOMAIN-like, NRT1/PTR FAMILY 5.2.*
  - 14 DEGs of JA signaling: *DELLA protein 3, DELLA4, EIN2-CEND, ethylene-responsive transcription factors, jasmonate-zim-domain proteins, Jasmonic acid-amido synthetase, transcription factor bHLH14, NRT1/ PTR FAMILY 5.2, TIFI*, and *bHLH*.

Overall, 45 transcription factors were differentially expressed *in vitro* and *in vivo*, with 14 TFs displaying different regulations. The following TFs were upregulated in both experiments: *HD-Zip, ERF2, R2R3-MYB anthocyanin 2, myb-related protein B-like, ERF2, B456_005G093000, mab-3-related, RADIALIS-like, WRKY40, WRKY53*, another four *WRKYs*, *RLP12, bHLH25 like, Scarecrow-like, ARF-like, TF Y subunit A-10-like*, two *NAC domain-containing proteins, nam-like protein*, and three unknown TFs (most of them annotated in *Actinidia sinensis* and *Camellia sinensis*). Another 14 TFs (*ZHD1, WOX4, NAC072, WRKY2-1, bHLH78, bHLH35, PPLZ02, MYB5c, CCCH Znf-*like, *HD-Zip-*like, *SBP-*like, *NAC-*like, *ERF-*like, and *AP2/ERF*-like) were upregulated *in vitro*, but downregulated *in vivo* except for *AP2/ERF* (Supplementary File S5).

Among the common significant DEGs, the following TFs were related to defense response to fungus and chitin (GO:0050832 GO:0010200): six *WRKYs* (TEAK023179, TEAK022875, TEAK009699, TEAK038999, TEAK011184, and TEAK034322), TEAK034291 (*Protein ENHANCED DOWNY MILDEW-like* [*Actinidia chinensis*]), TEAK002256 (*ethylene-responsive element-binding factor* [*Camellia sinensis*]), TEAK037695 (*ethylene response factor 2 (mitochondrion)* [*Camellia sinensis*]), TEAK000825 (unnamed protein product [*Coffea canephora*]), TEAK004458 (*NAC domain-containing protein* [*Actinidia chinensis* var. *chinensis*]), TEAK016352 (PREDICTED: *NAC domain-containing protein 90* [*Prunus mume*]), and TEAK005882 (*bHLH5* [*Diospyros kaki*]). In addition, several TFs were related to GO:0009611 (response to wounding) and GO:0050691 (regulation of defense response to virus): TEAK009699, TEAK038999 (*WRKY*), TEAK011184 (*WRKY40*), and TEAK039651 (*nam-like protein*).

Finally, 31 DEGs (14 downregulated) were related to the secondary metabolism and 112 DEGs (33 downregulated) were related to the cell wall metabolism. Among the genes related to the secondary metabolism, 15 genes belonged to the Cytochrome P450 family, with seven downregulated (*Cytochrome P450 711A1-*like, *71A26-*like, *78A5-*like, *78A5-*like, *81E8-*like, *81F3-*like, and *CYP736A12-*like) and eight upregulated (*cytochrome P450 71A4-*like, *71A6-*like, *81D11, 82A3-*like, *82C4-*like, *82C4-*like, *83B1-*like, *and CYP736A54*). The following genes of secondary metabolism were upregulated: three *DEAD-box ATP-dependent RNA helicases*, two *Flavonoid 3'-monooxygenases*, one *Premnaspirodiene oxygenase*, one *serine carboxypeptidase 1*, and an uncharacterized protein LOC112014040. Among the cell wall metabolism DEGs, several *cellulose synthases* (TEAK027889, TEAK017554, TEAK008472, and TEAK000831) and several *chitinases* (TEAK022306, TEAK018103, TEAK011494, TEAK011492, and TEAK034356) were upregulated. Seven of fifteen germin-like protein DEGs were also upregulated (TEAK021643, TEAK021642, TEAK035579, TEAK040734, TEAK040736, TEAK002466, and TEAK023261), ten *peroxidase* genes (four downregulated: TEAK036945, TEAK036946, TEAK025759, and TEAK031014; six upregulated: TEAK006197, TEAK006196, TEAK003744, TEAK009557, TEAK016084, and TEAK006195), five upregulated *Protein trichome birefringence-*like genes (TEAK036308, TEAK011619, TEAK028137, TEAK006259, and TEAK010627), four upregulated *Subtilisin*-like protease genes (TEAK027764, TEAK033538, TEAK027756, and TEAK002257), seven upregulated *Wall-associated receptor kinases* (TEAK017891, TEAK016034, TEAK016019, TEAK016022, TEAK017883, TEAK017870, and TEAK040051), and three upregulated *Beta-galactosidases* (TEAK009474, TEAK014932, and TEAK014217) (Supplementary Files S4 and S5).

**Figure 5:**
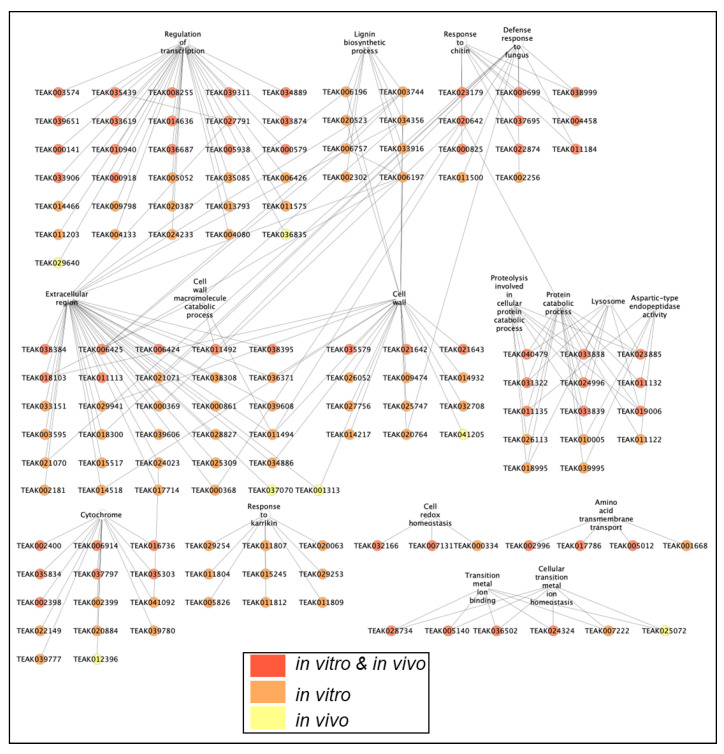
Genes associated with common GO terms for *in vivo* and *in vitro* ND response, transcription factors, and cytochrome genes, which are significantly upregulated with log_2_(FC) > 1. Expression patterns are marked by colors: red for both *in vitro* and *in vivo* experiments; orange for *in vitro*; and yellow for *in vivo*.

**Figure 6:**
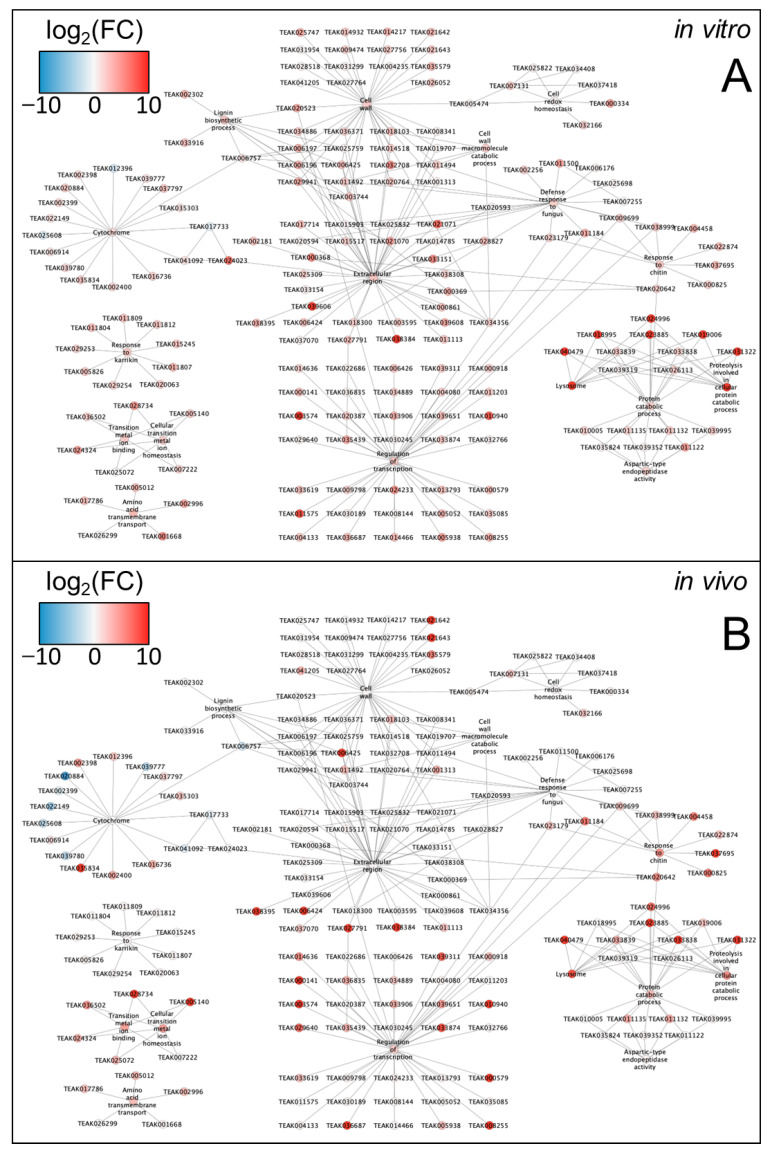
Fold changes of 175 genes from 15 common GO terms involved in nitrogen deficiency *in vitro* (**A**) and *in vivo* (**B**). The color indicates fold change in the log_2_-scale for genes and median value for GeneOntology terms, transcription factors, and cytochrome genes.

Gene networks were reconstructed to reveal and clarify relationships among genes associated with common GO terms for *in vivo* and *in vitro* ND response. First, the network combined significantly upregulated transcription factors, cytochrome genes (CYPs), and other DEGs from the common significantly enriched terms (143 genes out of 175 genes in total) (Figure 5). Among them, 61 genes were upregulated in both conditions, 75 genes were upregulated *in vitro* only, and 7 genes were upregulated *in vivo* only. Thus, *in vitro* conditions caused a predominant increase in the expression of genes from these categories. The upregulation of lignin biosynthesis genes, strigalactone response genes (response to Karrikin, etc.), and cell wall genes was much more pronounced under *in vitro* conditions. Detailed gene networks of 175 genes from 15 common GO terms were reconstructed for *in vitro* (Figure 6A) and *in vivo* (Figure 6B) experiments. The results showed that *EG45-like domain protein* (TEAK24023) from the extracellular region (GO:0005576) interacts with two *cytochromes* (TEAK017733 and TEAK041092). Additionally, *cytochrome-like protein* (TEAK006757) was associated with the biosynthesis of lignin, three *cell wall peroxidases* (TEAK006197, TEAK003744, and TEAK025759), and *berberine bridge enzyme-like protein* (TEAK006425). We also found that three *glutaredoxins* (TEAK025822, TEAK034408, and TEAK037418), *peroxiredoxin* (TEAK005474), and *cytosolic glutathione reductase* (TEAK007131) were upregulated under ND. These results further clarify the signaling mechanisms and regulation of the response to ND in *C. sinensis*.

## 3. Discussion

The *in vitro* approach is known for its advantages, such as strictly controlled growth conditions and simpler intracellular organization. Plants cultivated *in vitro* can serve as an informative model to study the plant responses to different stimuli at the cell and tissue level. In this study, we used an *in vitro* vs. *in vivo* transcriptomic approach to reveal common responsive pathways under nitrogen deficiency in tea plant. The experimental plants *in vitro* were without a root system and displayed different ND responses as compared to potted plants. Particularly, L-theanine content, which is an important component of tea leaf quality, was low in vitro in both control and ND-treated plants. This may be due to the absence of roots in these plants, as it is well known that L-theanine serves as a nitrogen pool and is synthesized in roots and then transferred and accumulated in the young shoots [27,28,29]. In contrast, ND resulted in strong inhibition of leaf L-theanine content *in vivo*. Additionally, several catechins displayed less pronounced ND response *in vitro* as compared to *in vivo*, where an increased accumulation of EC, GC, Catechin, and EGC was observed. On the other hand, the accumulation of catechin gallates was less affected by experimental conditions. Usually, L-theanine and catechins are negatively correlated with each other. Nitrogen utilized by roots is usually transformed into N storage amino acids such as L-theanine, which can further convert it into secondary metabolites, such as simple catechins [30]. This may be the reason why catechin content was more affected by ND *in vivo* as compared to *in vitro*. Surprisingly, methylxanthines (theobromine and caffeine) were more strongly inhibited by *in vitro* ND as compared to *in vivo* ND, suggesting their dependence on the presence of roots.

When we analyzed the RNA-seq results, we mostly focused on the ND responses that were common for both experiments to reveal the core responsive pathways. The most enriched common GO terms *in vitro* and *in vivo* were related to redox processes, cell wall remodeling, and phenylpropanoid and secondary metabolism pathways. Additionally, amino acid transport and response to Karrikin were enriched, which is consistent with the earlier ND studies [31,32]. The reconstructed gene network revealed abundant connections of the lignin biosynthesis DEGs with the other DEGs, indicating the important role of lignin biosynthesis for ND response in tea plant, which is consistent with the other studies [33,34,35]. These DEGs were closely associated with several cytochromes playing a key role in flavanol biosynthesis. The reconstructed gene network displayed that *cytochrome-like protein* (TEAK006757) is associated with the biosynthesis of lignin, three *cell wall peroxidases* (TEAK006197, TEAK003744, and TEAK025759) and *berberine bridge enzyme-like protein* (TEAK006425). We also found that *EG45-like domain protein* (TEAK24023) from the extracellular region (GO:0005576) interacts with two *cytochromes* (TEAK017733 and TEAK041092). These results are consistent with a recent study that reported the important role of *CYPs* in the adaptation to ND in other crops [36] and clarify the signaling and adaptation mechanisms of the response to ND in *C. sinensis*. CYPs play a decisive role in the biosynthesis of secondary metabolites, antioxidants, and phytohormones in higher plants [37]. It was previously shown that cytochromes and peroxidases were involved in the response to early ND in cucumber and these proteins regulate flavonoid biosynthesis [38]. According to recent studies, CYPs participate in crosstalk between abiotic and biotic stress responses and have enormous potential to be used as candidates for engineering tolerance to biotic and abiotic stresses [39].

Under ND, most DEGs of the MAPK signaling pathway and ABC transporters were significantly upregulated *in vitro* and *in vivo,* and most downstream DEGs were related to calcium signaling rather than hormone signaling. Recent studies reported that the MAPK signaling pathway takes an active part in the transmission of nuclear signals, since the majority of MAPK targets are transcription factors [40,41]. Most of the hormone signaling DEGs were related to auxins and ABA, indicating the crucial role of these hormones in ND response. It is known that shoot tips and leaves are the main organs for auxin biosynthesis while roots are the main organ for cytokinin biosynthesis [42]. Thus, in leaf mRNA samples, we observed many DEGs related to auxin signaling in both experiments. According to a recent study, the auxin signaling pathway controls root hair formation, which is activated under ND conditions as an adaptation for better absorption of soil nitrogen [43]. Reconstructed gene networks displayed that three *glutaredoxins* (TEAK025822, TEAK034408, and TEAK037418), *peroxiredoxin* (TEAK005474), and *cytosolic glutathione reductase* (TEAK007131) were upregulated under ND. Apparently, these components of the antioxidant system mediate plant response and signaling, as was previously demonstrated for *CC-type glutaredoxins* in *A. thaliana* under ND [44]. Additionally, individual transporters and enzymatic components of the response to Karrikin are also associated with the response to abiotic stress. Particularly, *raffinose synthases* plays a role in the response to drought in dicotyledonous and monocotyledonous plants [45], suggesting that it is an important mechanism common to different stress responses. These results clarify the signaling mechanisms and regulation of response to nitrogen deficiency in evergreen woody species.

According to both KEGG and GO analysis, pathways related to photosynthesis were enriched *in vitro* and *in vivo* under nitrogen deficiency. Many downregulated DEGs were associated with the Photosystem I reaction center (GO:0009538) and Photosystem I light-harvesting complex (GO:0016168), which is consistent with recent studies in orange [46], blueberries [47], and tea plant [22]. We suggest that an increased expression of *ELIP2* resulted in chlorophyll degradation and further remobilization of chloroplast nitrogen to secondary metabolism (Figure 3). This is consistent with an earlier study on wheat, which reported the remobilization of chloroplast nitrogen under ND, resulting in photosynthesis inhibition [48]. It was earlier reported that the physiological function of *ELIP* is related to the regulation of chlorophyll content in thylakoids: *ELIP* genes work as chlorophyll sensors, modulating chlorophyll synthesis, preventing the accumulation of free chlorophyll and therefore preventing photo-oxidative stress in Arabidopsis [49]. In tea leaves, two *CsELIP* genes (*ELIP1* and *ELIP2*) were significantly upregulated when photoinhibition occurred, which means they may be involved in photoprotection [50]. Our recent mRNA-seq study showed that *CsELIP* expression is elevated one hundred-fold in tea leaves under long-term cold, freezing, and drought [51]. Thus, ELIP genes can be very important in response to multiple environmental stresses including ND.

Overall, 45 transcription factors were upregulated in both *in vivo* and *in vitro* experiments, five *WRKYs*, three *NACs*, two *MYBs*, two *ERFs*, *HD-Zip*, *RLP12*, *bHLH25*, *RADIALIS*-like, and others, suggesting their ND regulation is independent from the presence of a root system. Most transcription factors belong to ABA-mediated stress response (*bHLH, MYB, WRKY, bZIP, NAC*, etc.) which is consistent with the ND response of wheat [52] and blueberry [47]. Gene network reconstruction displayed that these TFs were related to the response to fungus/chitin, suggesting that nitrogen response and pathogen response have common regulation. These TFs families have been reported to play a crucial role in multiple responses to abiotic and biotic stresses [53,54,55]. Particularly, members of the *WRKY*, *bHLH*, *MYB*, and *bZIP* families were involved in tea plants in cold, salt, and drought responses [56,57]. It is known that *MYB* TFs interact with *bHLH* and form the *MYB-bHLH* complex, which involved in the synthesis of phenylpropanoids, cell wall remobilization, and protection from phytopathogens [47,53,58]. Transcription factor *WRKY53* was found to be an important regulator of the senescence process in *A. thaliana* [59]. *BHLH87*, the known regulator of epigallocatechin-3-gallate biosynthesis, was significantly downregulated in *C. sinensis* in response to three ecological factors [60]. *BHLH25*-like in *C. sinensis* was significantly downregulated in conditions of aluminum stress [61]. Overexpression of the cotton *GhNAC072* gene enhances drought and salt stress tolerance in *A. thaliana* [62]. Thus, the common biological processes associated with persistent changes in the transcriptome demonstrate a pattern of involvement of both general metabolic and very specific components in the response to nitrogen deficiency. Finally, many TFs (*ZHD*1, *WOX4*, *NAC072*, *WRKY2-1*, *bHLH78*, *bHLH35*, *PPLZ02*, *MYB5c*, *CCCH Znf*-like, *HD-Zip*-like, *SBP*-like, *NAC*-like, and *ERF*-like) were upregulated *in vitro*, but downregulated *in vivo*, suggesting their regulation is dependent on the presence of the root system.

## 4. Materials and Methods

### 4.1. Plant Material and Stress Induction

The plant material was taken from the collection of the Subtropical Scientific Centre, Sochi, Russia. This study was carried out in 2022–2024.

For the *in vitro* experiment, micropropagated plantlets of diploid cv. Kolkhida (*Camellia sinensis* L.) were used. This large-leaf diploid tea cultivar, characterized by perfect tea leaf quality and good yield, was derived in the USSR. These plantlets were propagated vegetatively on half Murashige and Skoog (MS) nutrient medium, under the following conditions: a photoperiod of 16/8 h, temperature of 25 ± 1.0 °C, humidity of 70%, and illumination of 3000 lux (tubes L 36 W/765, «OSRAM GmbH», Munich, Germany). For the ND experiment, plantlets with a height of 2.0–3.0 cm with 3–5 fully developed leaves without a root system were randomly selected. ND was induced by completelu excluding nitrogen-containing compounds (KNO_3_, NH_4_NO_3_, and casein hydrolysate) from the nutrient medium, while the control treatment contained full-composition MS. Each treatment (ND and control) included 30 plantlets (10 plantlets for each biological replicate). The leaf sampling was conducted after 2 months of the experiment. Three biological replicates were used, each of them representing a mixed leaf sample of 5 plantlets (for biochemical analysis) and 3 plantlets (for RNA-seq and qRT-PCR).

For the *in vivo* experiment, healthy vegetatively propagated 3-year-old plants of tea cv. Kolkhida were cultivated in 4 L pots filled with clean river sand, with three plants with three replications per treatment. Seven days after subculture, these plants were watered with 50% nutrient solution (pH 5.0–5.1) of the following content: 3 mM NH_4_NO_3_, 0.5 mM Ca(H_2_PO_4_)_2_, 1.0 mM K_2_SO_4_, 0.5 mM CaCl_2_, 0.6 mM MgSO_4_, 46 µM H_3_BO_3_, 2 µM CuSO_4_, 9 µM MnSO_4_, 2.6 µM Na_2_MoO_4,_ 30 µM Fe-EDTA, and 9 µM ZnSO_4_. After 14 days, the experimental plants were watered every two days with 500 mL of the 100% nutrient solution (treatment N0) or 3 mM NH_4_NO_3_ (control N+) [63]. During the whole experiment, the plants were maintained at the open-roof greenhouse under the shedding with following conditions: a temperature of +24 ± 4 °C, natural light of 3000 ± 200 lux, and substrate water content of 70 ± 10%. The leaf samplings were conducted after 2 and 4 months of the experiment. Three biological replicates were used, each of them representing one plant; mature leaves (3rd–4th from the top) were sampled for biochemical and RNA-seq and qRT-PCR analyses.

### 4.2. Phenotyping of ND Response

The leaf nitrogen content was measured spectrophotometrically using the Kjeldahl method as previously described [64].

Tea leaves were fixed by steam treatment at 100 °C for 5 min in a water bath and then dried. The contents of caffeine, L-theanine, and catechins (mg g^−1^ dry leaf mass) were estimated by HPLC with methanol extraction according to the previously published protocol [64].

### 4.3. mRNA-Sequencing, DEG Identification, and Gene Annotation

RNA libraries were constructed using standard Illumina protocols and RNA sequencing was performed by Novogene Co. Ltd (Cambridge, UK). using the Illumina Hi-Seq platform. Read quality control was performed using fastp with read alignment with HIS AT2 [65]; .bam files were produced with SAMTOOLS v.1.9 [66]. Gene expression was calculated with RSEM [67] and normalized by RPKM and significant DEGs were revealed using DEseq2 [68] with *p* value < 0.05, |log_2_FC| > 1, FDR < 0.1. Pheatmap (V1.0.8) [69] and Venn web (https://bioinformatics.psb.ugent.be/webtools/Venn/, accessed on 27 October 2024), were applied for visualization, and Clusterprofiler (https://bioconductor.org/packages/clusterProfiler/, accessed on 27 October 2024) was used for GO (http://www.geneontology.org/, accessed on 27 October 2024) and KEGG (https://www.kegg.jp/, accessed on 27 October 2024) analyses.

We combined de novo, homology-based and RNA-seq methods to predict protein-coding genes in the tea plant cv. Kolkhida genome (https://db.cngb.org/search/?q=CNP0005366, accessed on 27 October 2024). For de novo prediction, AUGUSTUS [70] and GENESCAN [71] were applied. For the homology prediction, TBLASTN [72], solar (v.0.9.6) [73] (parameters “-a prot2genome2 –z”), and Genewise [74] were used with the following species: *Actinidia chinensis*, *Arabidopsis thaliana*, *Camellia oleifera*, *Camellia sinensis* cv TGY, *Vitis vinifera*, and uniprot_sprot_plants. For the RNAseq prediction, STAR (2.7.9a) [75] and String (v2.1.7) [76] (parameters “-j 2 -f 0.01 -c 2 -m 200 -a 10”) were used for the mapping and assembly of the transcripts. Finally, all results were combined by Maker (v.01.03v 3.01.03) [77], for annotation of protein-coding genes.

### 4.4. Verification of the RNAseq Results by qRT-PCR

The total RNA was extracted from leaves by Trizol (Biolabmix, Novosibirsk, Russia) and treated by DNase I (Biolabmix, Novosibirsk, Russia) with subsequent DNAse elimination by chloroform, with subsequent reprecipitation. The quality of RNA was checked by electrophoresis and spectrophotometry and cDNA samples were prepared using M-mulv reverse transcriptase (Biolabmix, Russia). The reference gene Actin (NCBI Gene ID: 114316878) was used for normalization of gene expression. The relative gene expression level was calculated using Livak and Schmittgen’s (2001) method as 2^−∆∆Cq^ [78]. The detailed protocols and PCR conditions are available in our recent study [51].

### 4.5. Data Analysis and Availability

Data were analyzed with the XLSTAT package (free trial version) (Lumivero, Denver, CO, USA https://www.xlstat.com/en/, accessed on 27 October 2024) by one-way ANOVA with multiple comparisons tests. Protein–protein interactions were reconstructed using String-db v. 12.0, the input option using proteins by sequences [79], and the type of interactions by experiments and databases, with a minimum required interaction score of 0.400. Visualization of GeneOntology networks was carried out using Cytoscape v. 3.9.1 software [80].

## 5. Conclusions

In this study, we used an *in vitro* vs. *in vivo* experimental design to reveal core ND-responsive mechanisms in *Camellia sinensis* (L.) Kuntze. We compared the ND response of *in vitro* plantlets (without a root system) and *in vivo* plants (with a root system). HPLC analysis revealed that most catechins were accumulated by tea leaves under ND *in vivo*, but not *in vitro*. RNA-seq and RT-qPCR data and GO and KEGG analyses revealed key enriched pathways under ND *in vitro* and *in vivo*. The reconstructed gene network revealed that cell wall metabolism and lignin biosynthesis DEGs present abundant connections with the other DEGs. We suggest that lignin biosynthesis is a crucial responsive pathway which is not mediated by the root system. This pathway was closely associated with several cytochromes which play key roles in flavanol biosynthesis in tea plant. Overall, 45 transcription factors were upregulated in both *in vivo* and *in vitro* experiments: five *WRKYs*, three *NACs*, two *MYBs*, two *ERFs*, *HD-Zip*, *RLP12*, *bHLH25*, *RADIALIS*-like, and others. Gene network reconstruction displayed that these TFs were related to fungal response, suggesting that nitrogen response and pathogen response have common regulation. Overall, the results clarify the signaling and regulation mechanisms under nitrogen deficiency in tea crop.

## Data Availability

Raw sequencing data are available at https://db.cngb.org/search/?q=CNP0004775, accessed on 27 October 2024 (transcriptomes) and https://db.cngb.org/search/?q=CNP0005366, accessed on 27 October 2024 (reference genome of cv. Kolkhida).

## Supplementary Materials

The supporting information can be downloaded at: https://www.mdpi.com/article/10.3390/ijms252111726/s1.
