# Supplementary figures and images for: In Vitro vs. In Vivo Transcriptomic Approach Revealed Core Pathways of Nitrogen Deficiency Response in Tea Plant (Camellia sinensis (L.) Kuntze)
Source: Int J Mol Sci. 2024 Oct 31;25(21):11726. doi: 10.3390/ijms252111726 (PMC11547157; doi:10.3390/ijms252111726)

#1-3 – Kolkhida ND in vivo

1

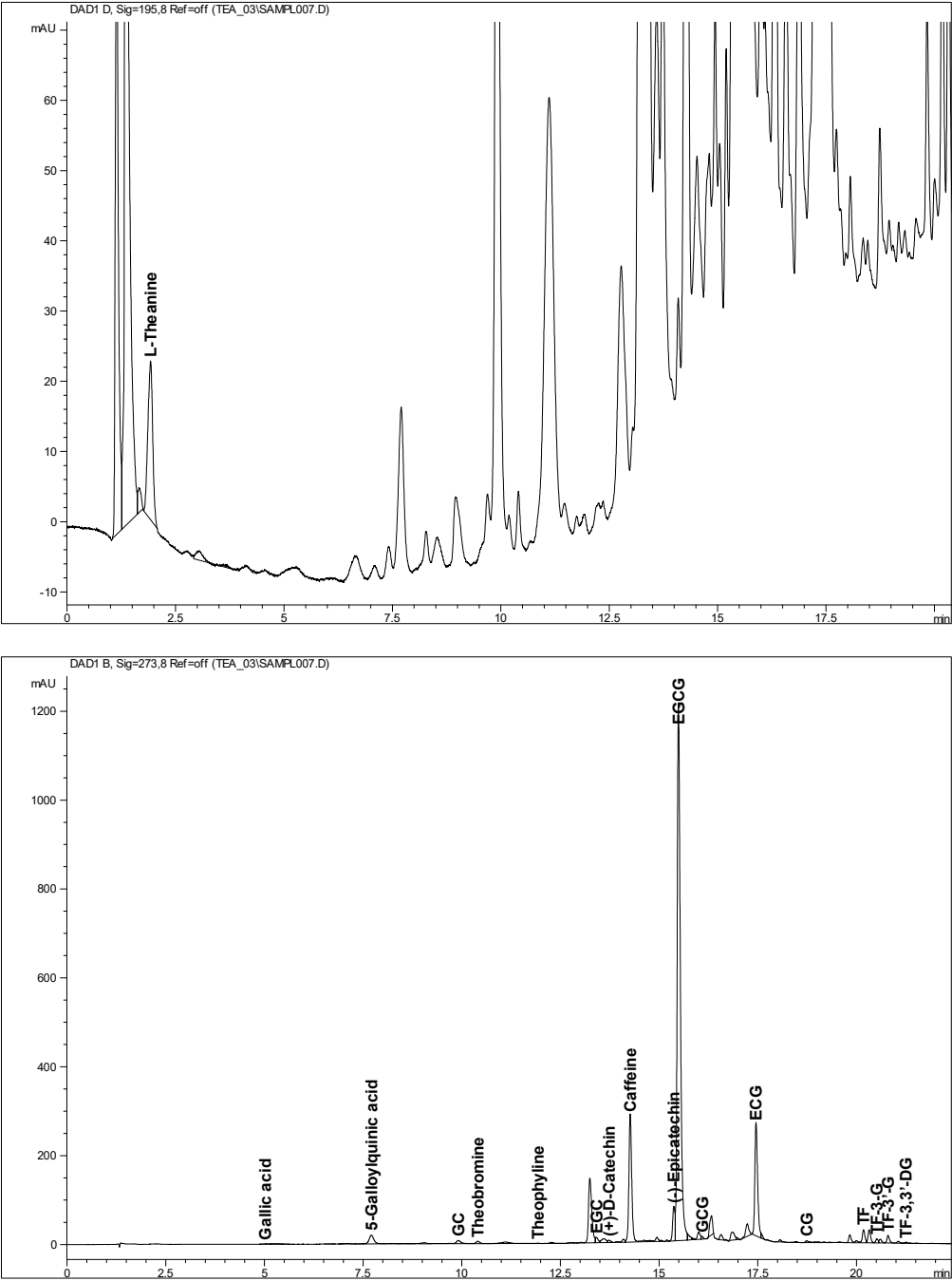

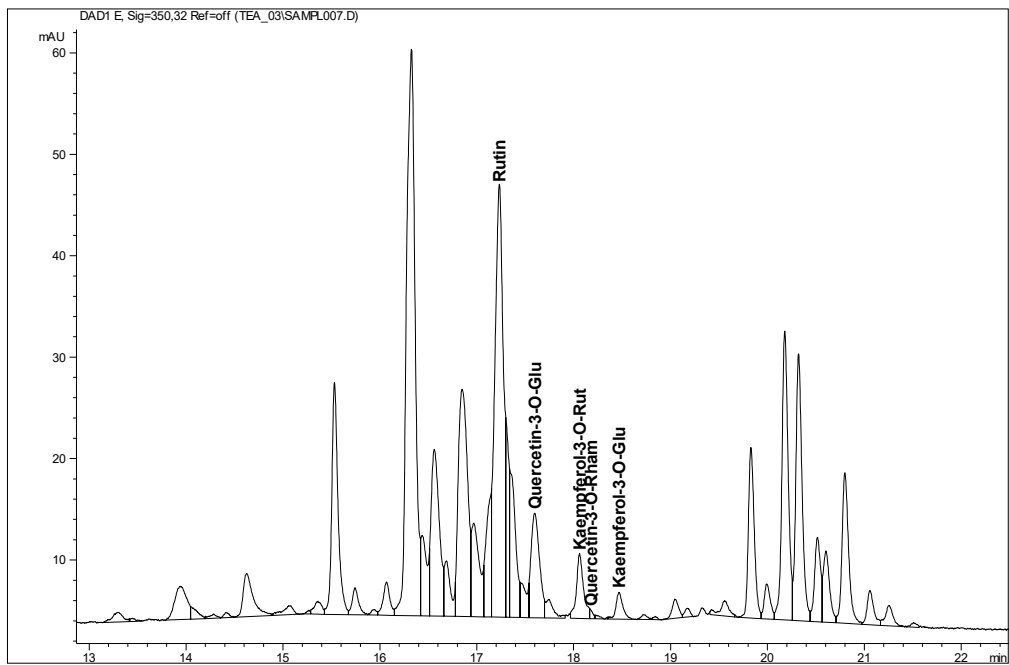

2

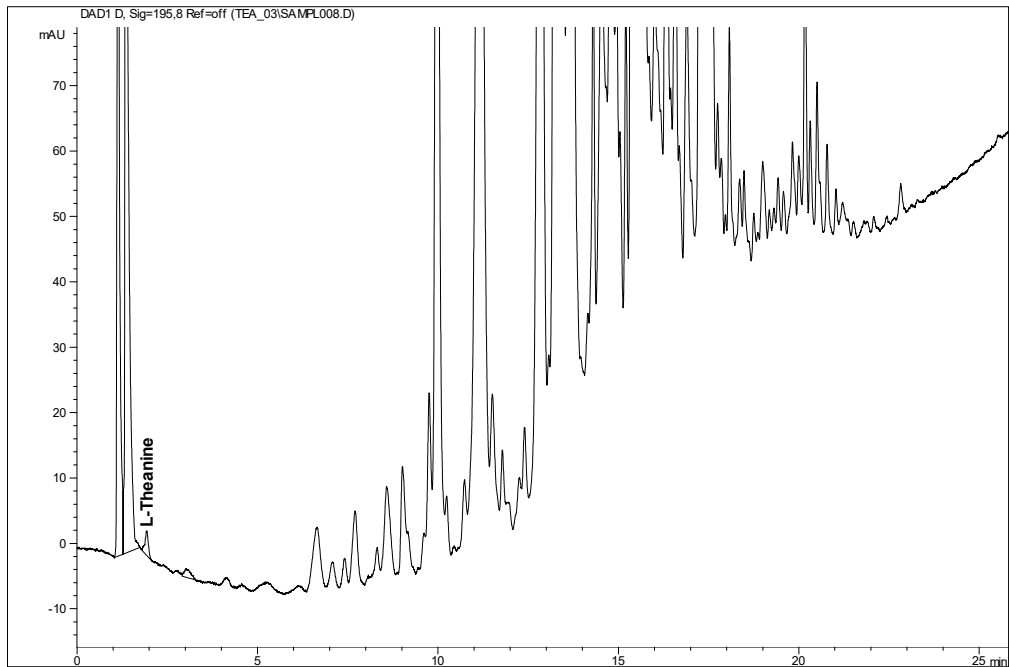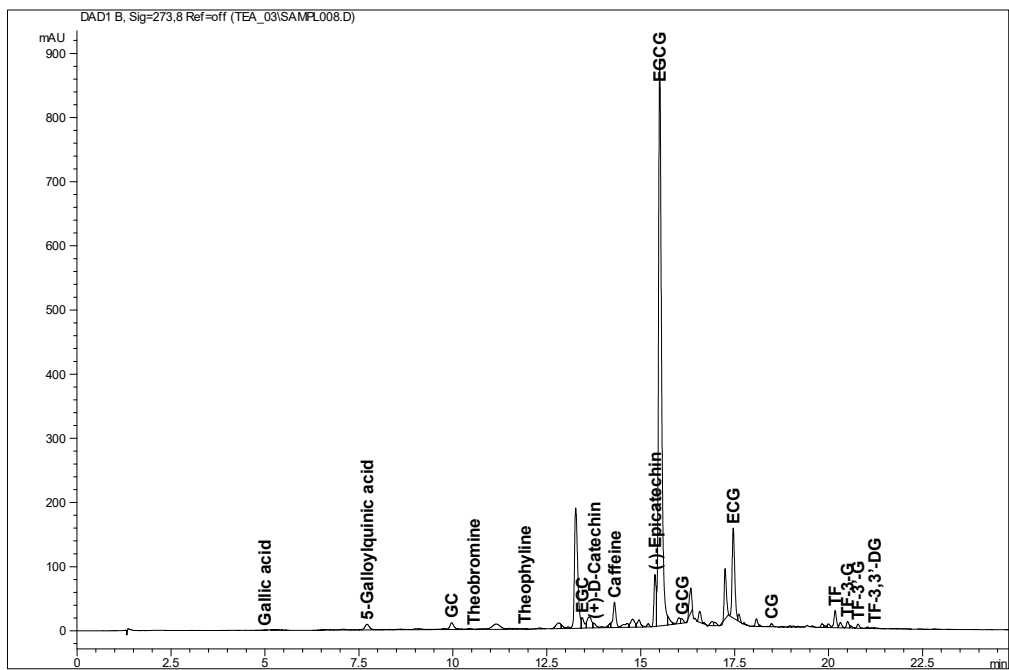

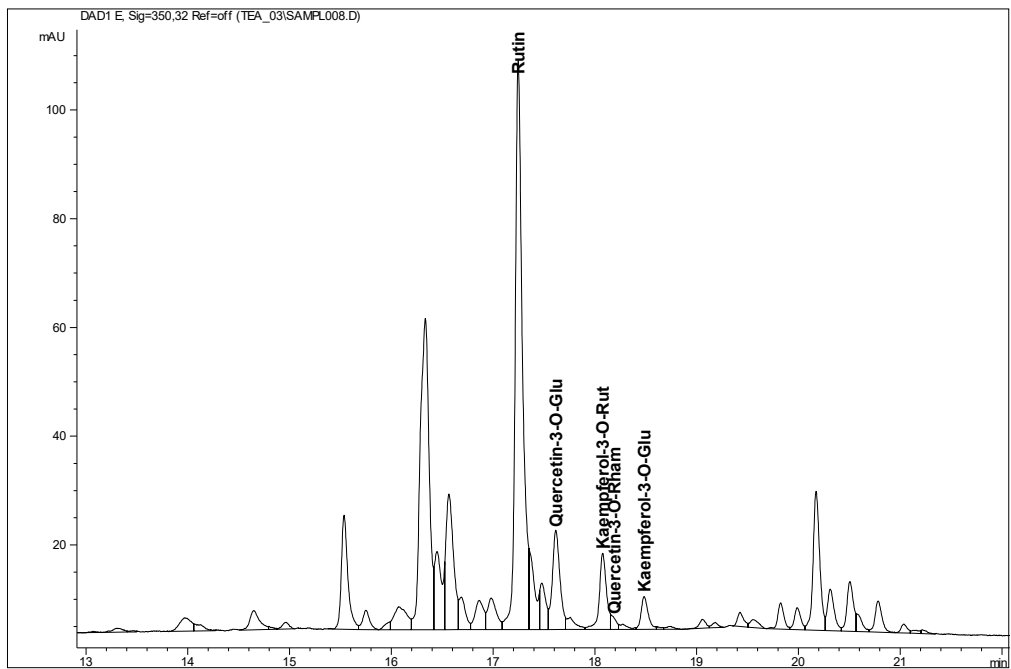

3

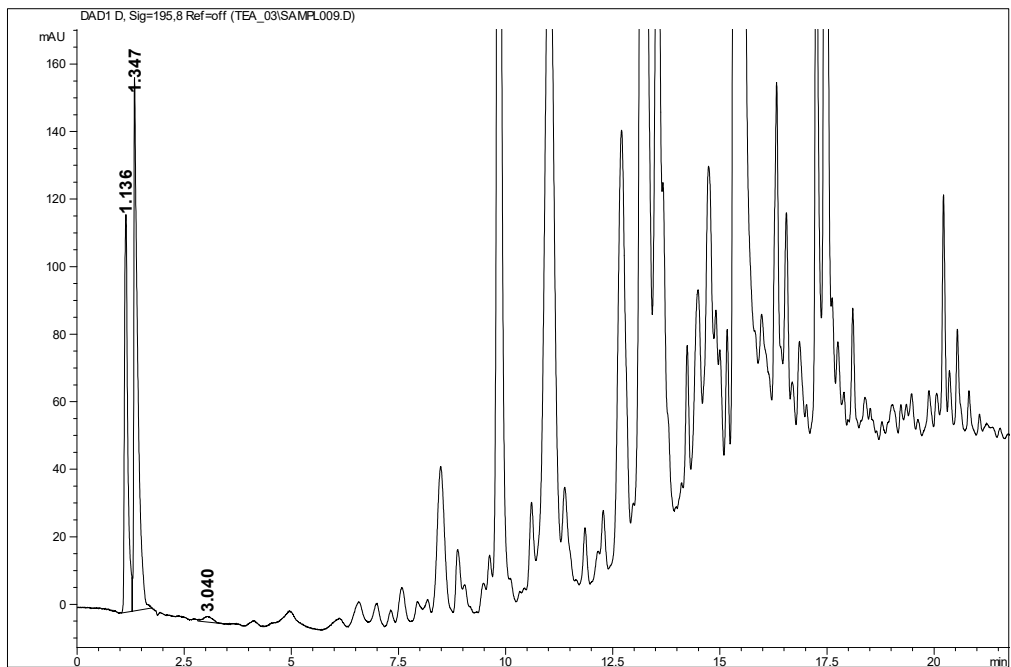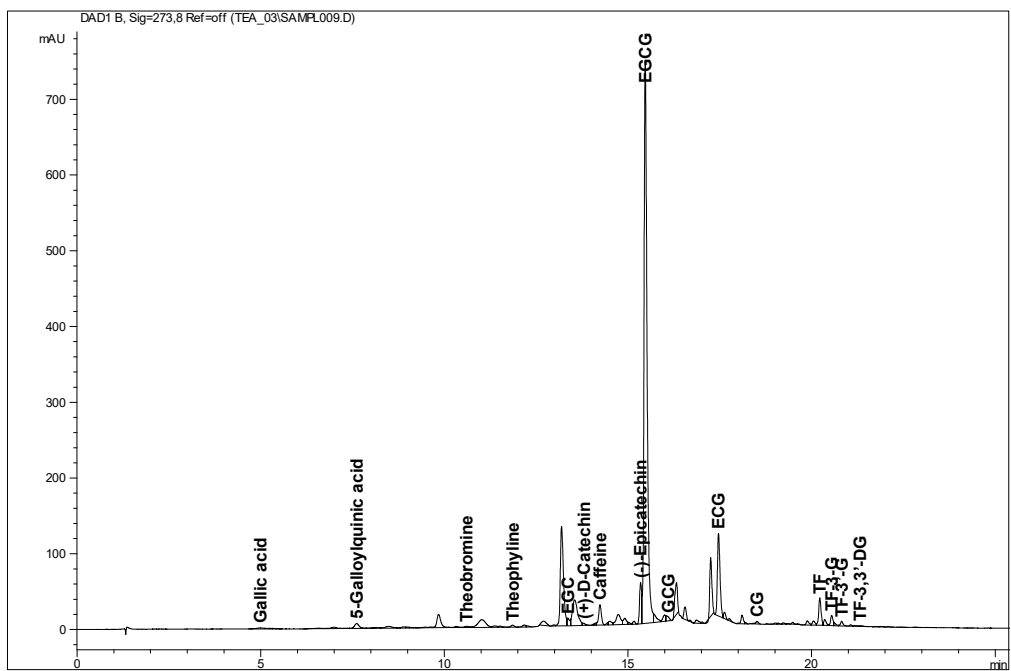

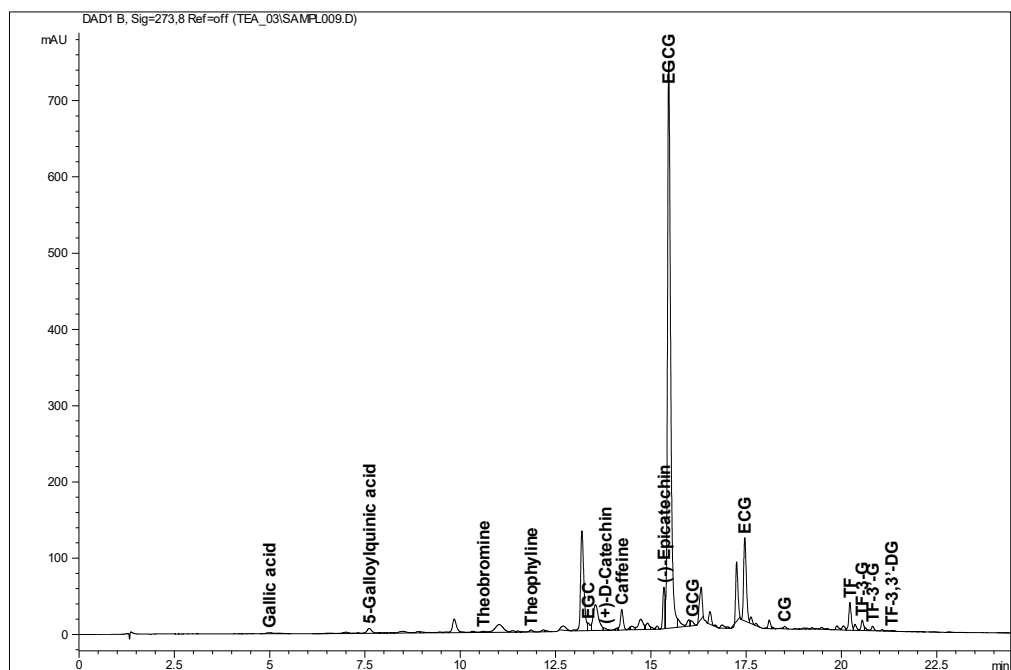

## #4-6 – Kolkhida N+ in vivo

4

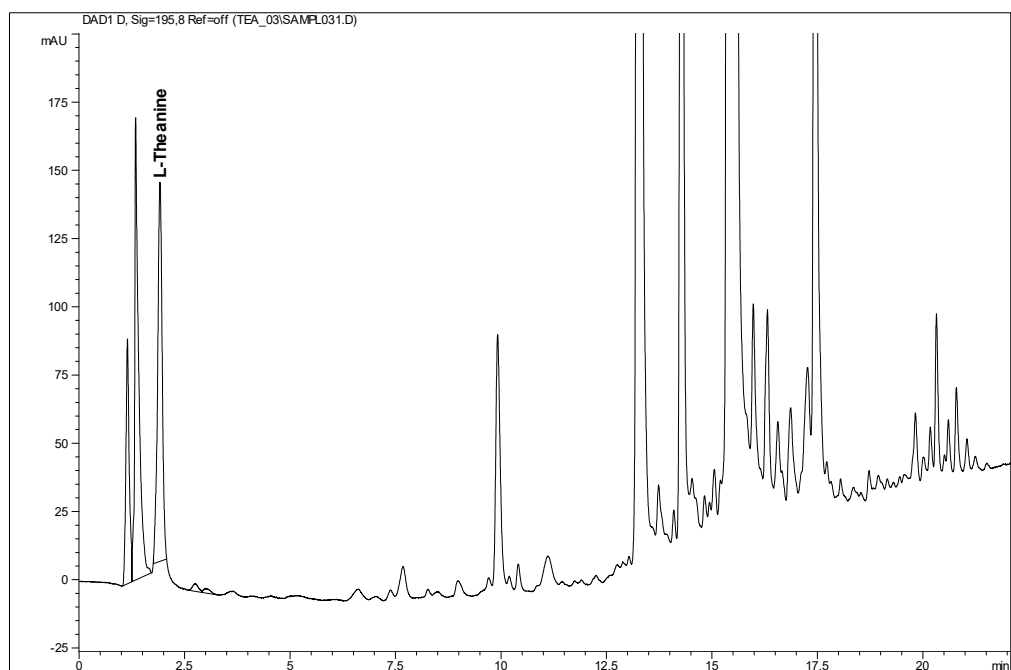

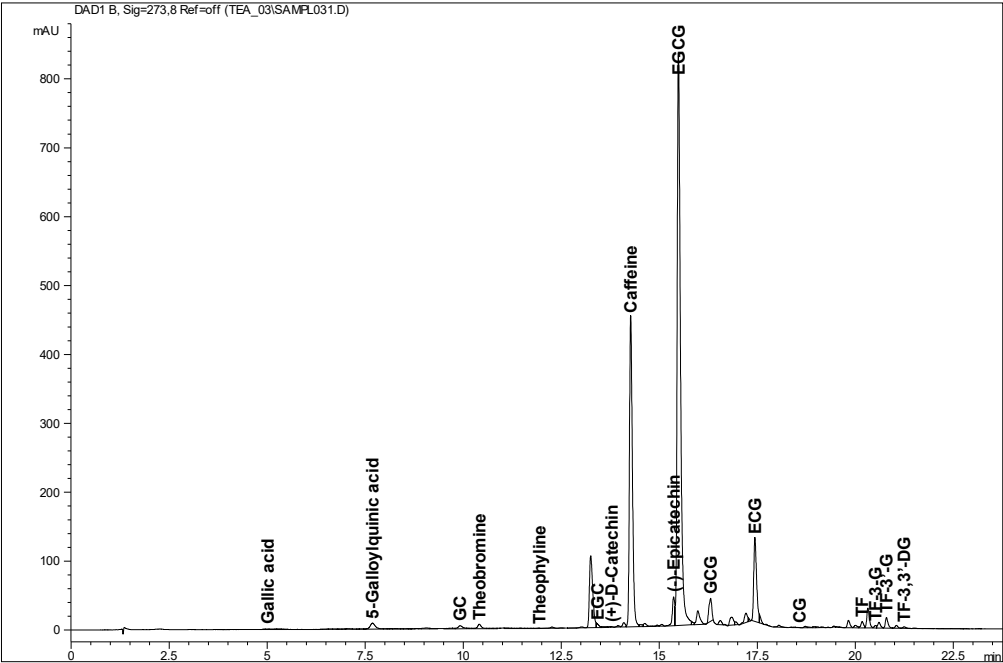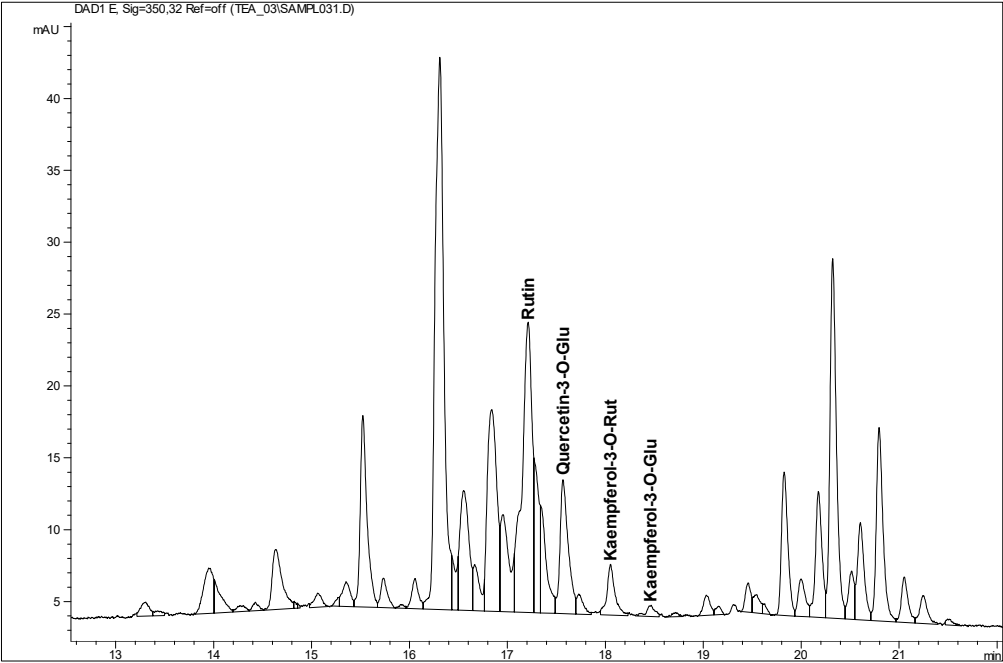

5

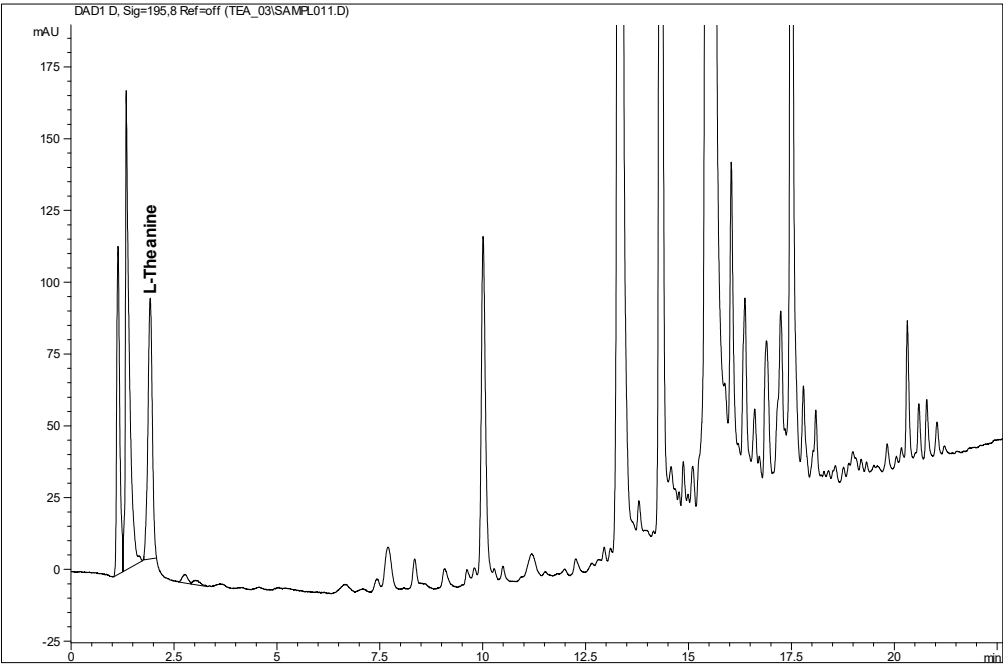

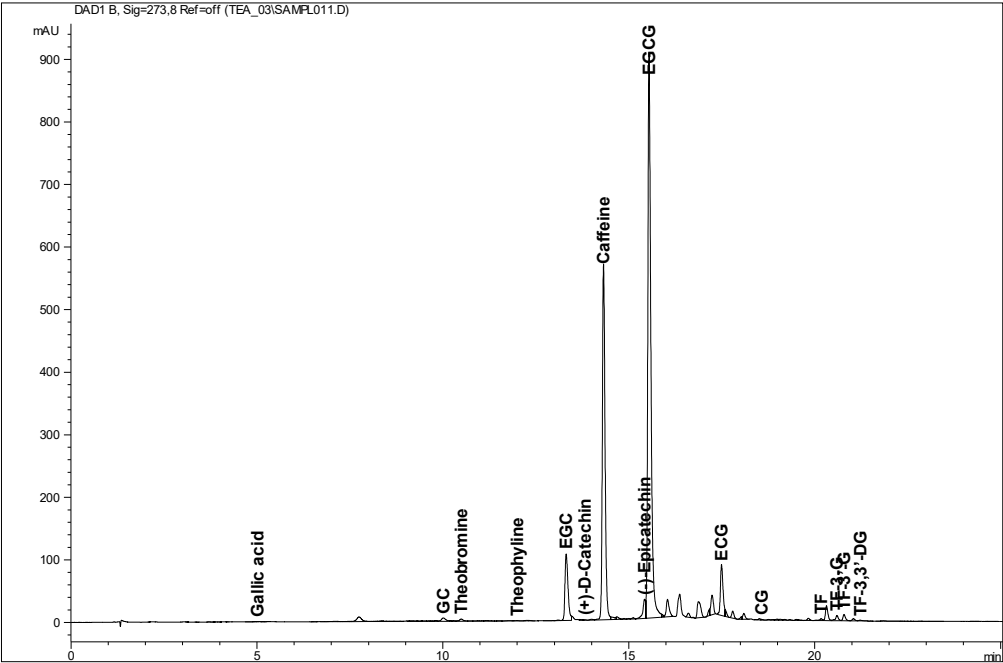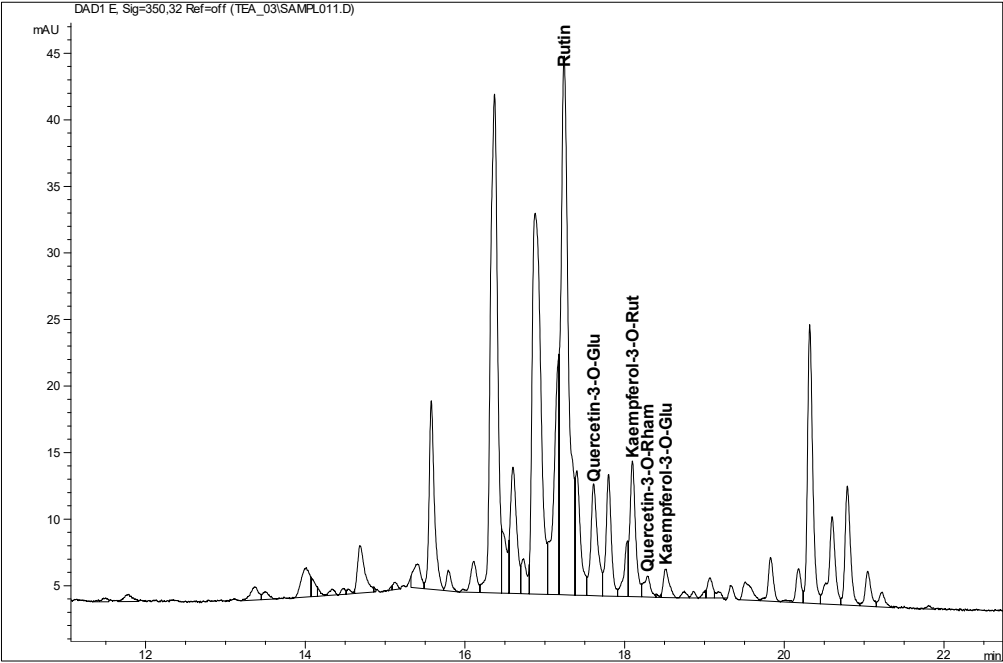

6

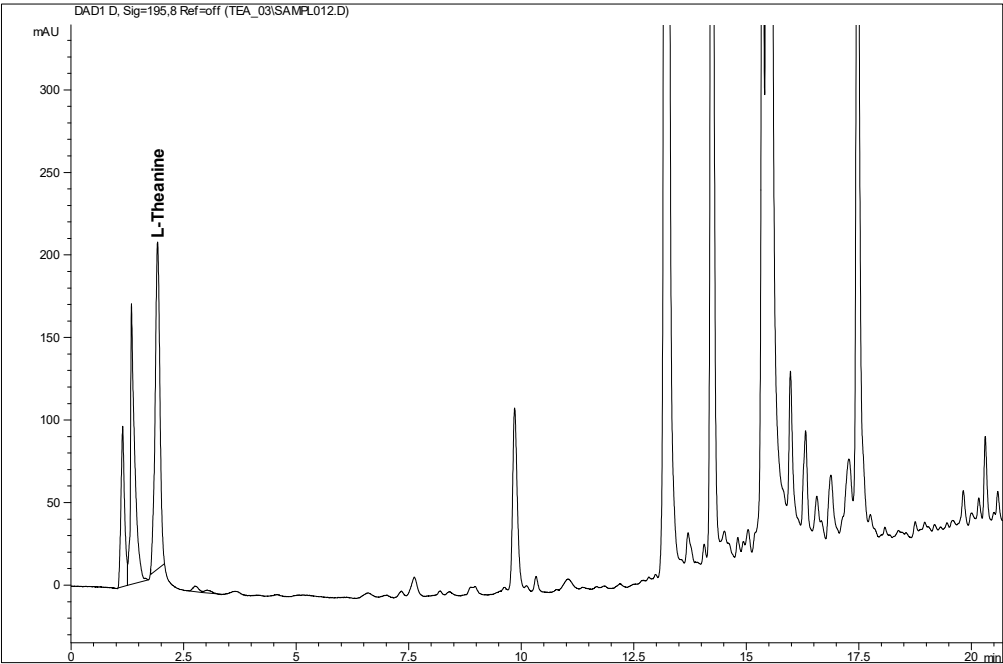

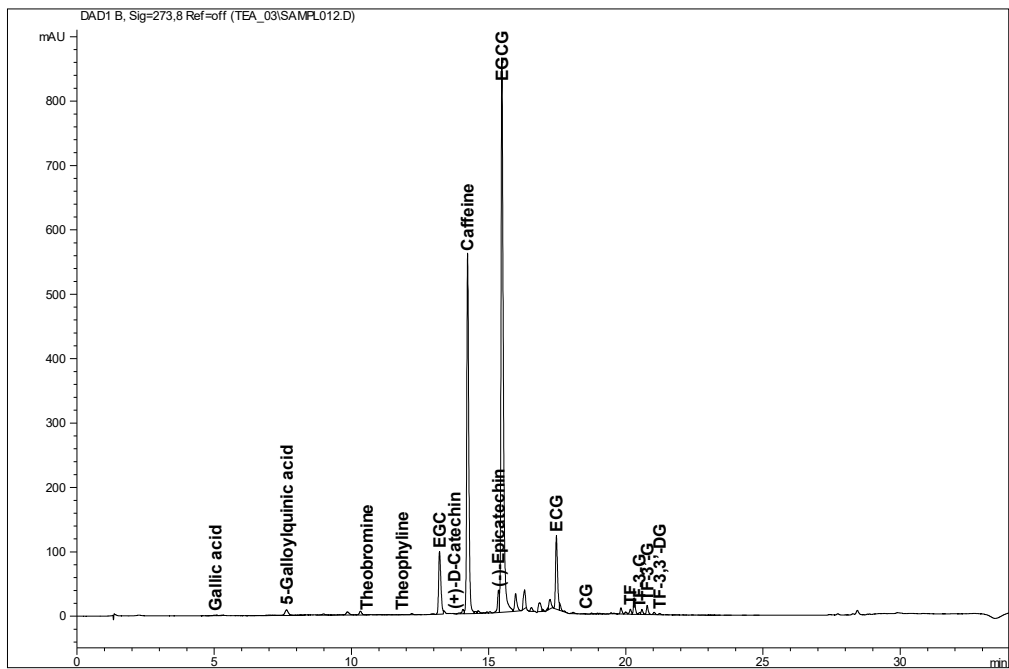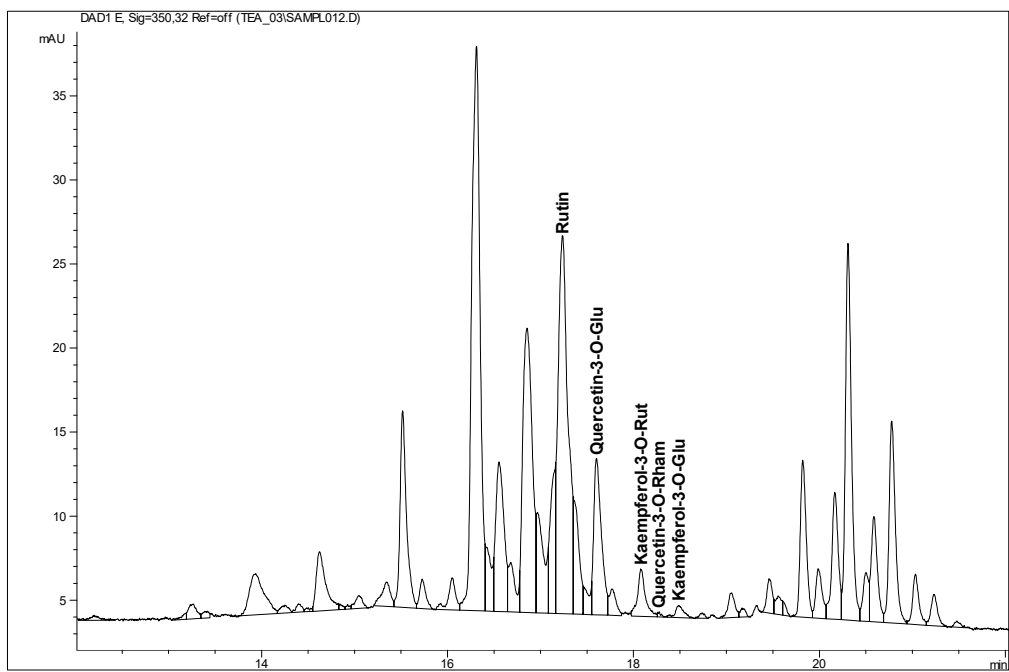

**#7-9 Kolkhida ND in vitro**

**7**

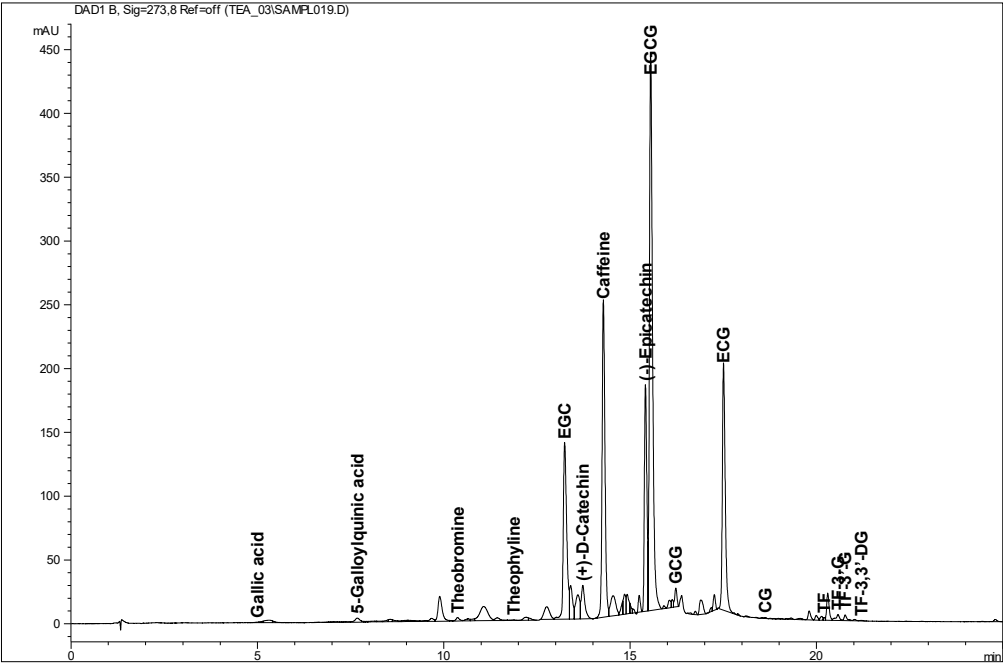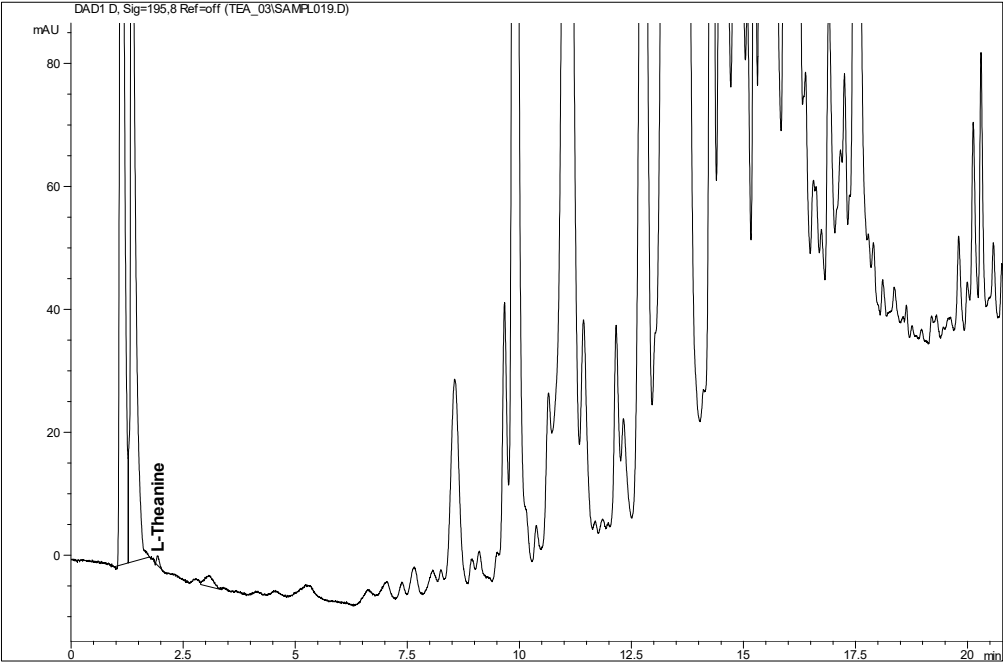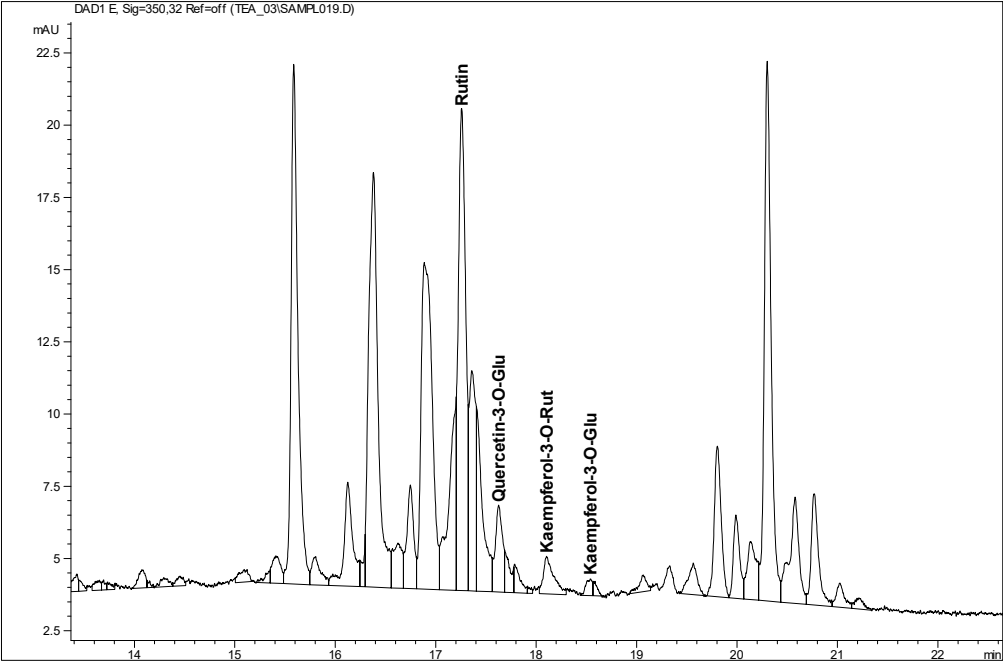

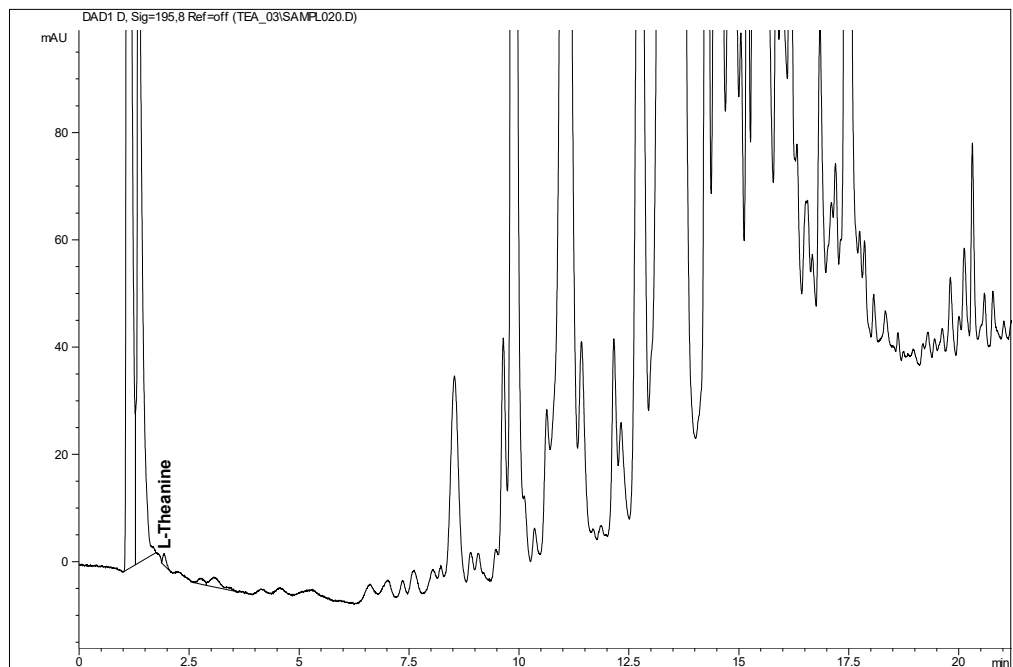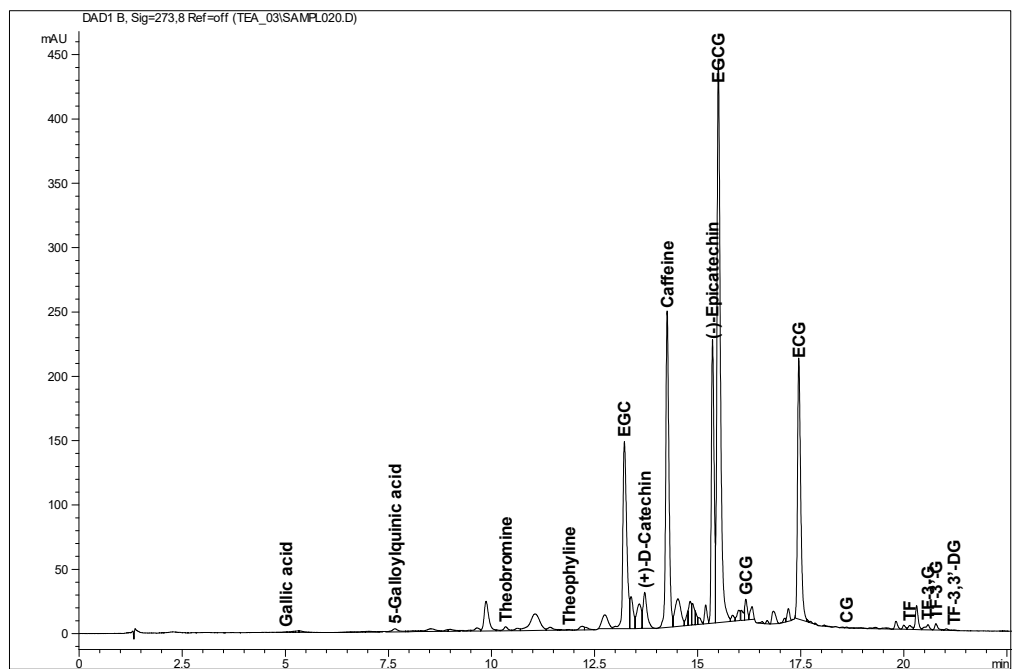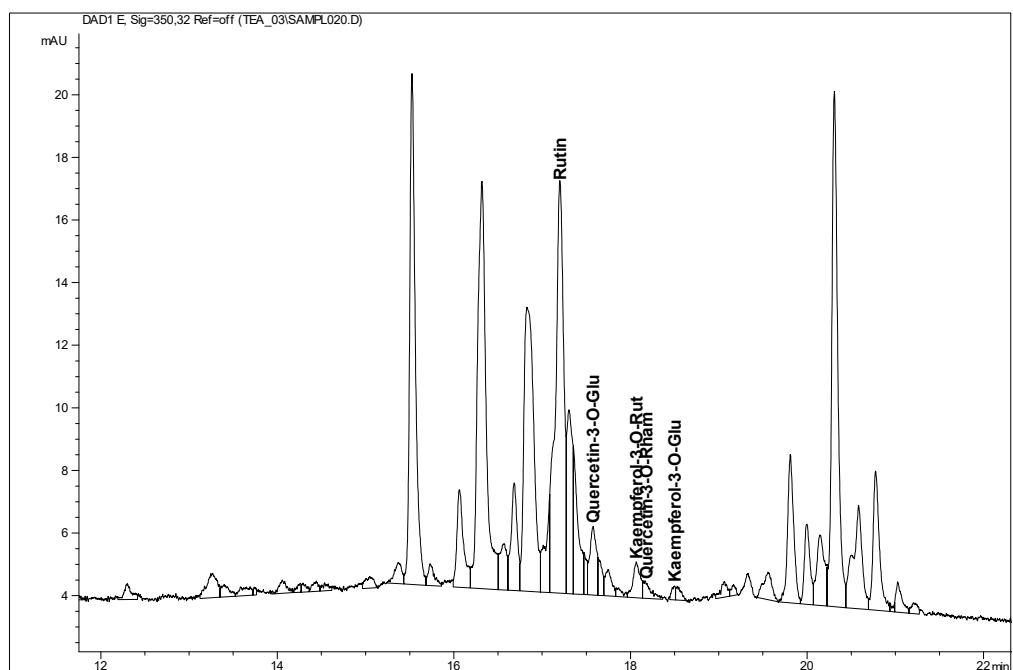

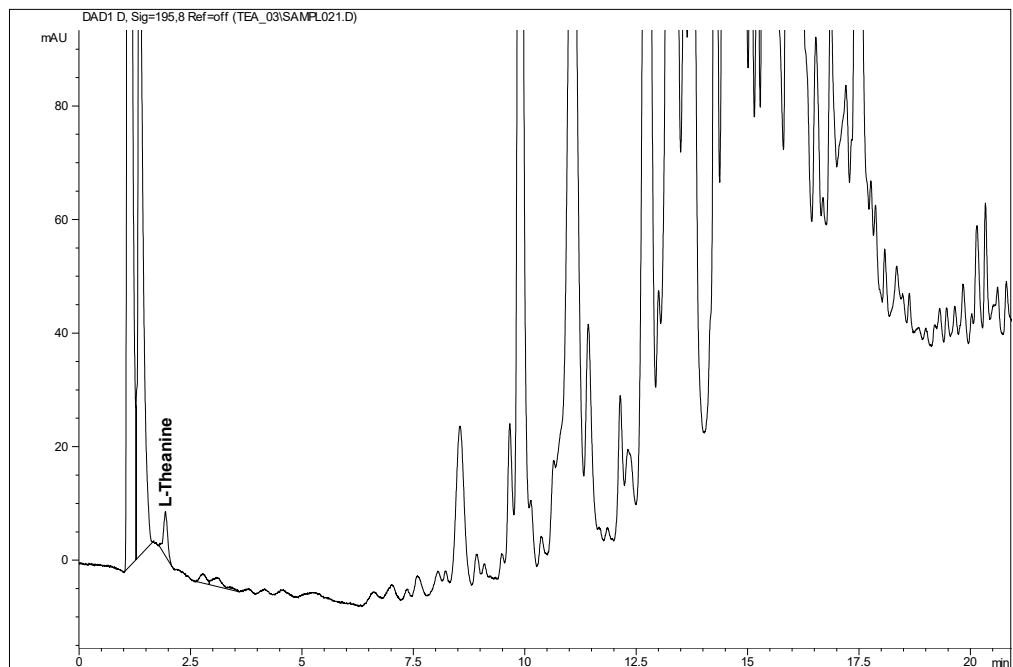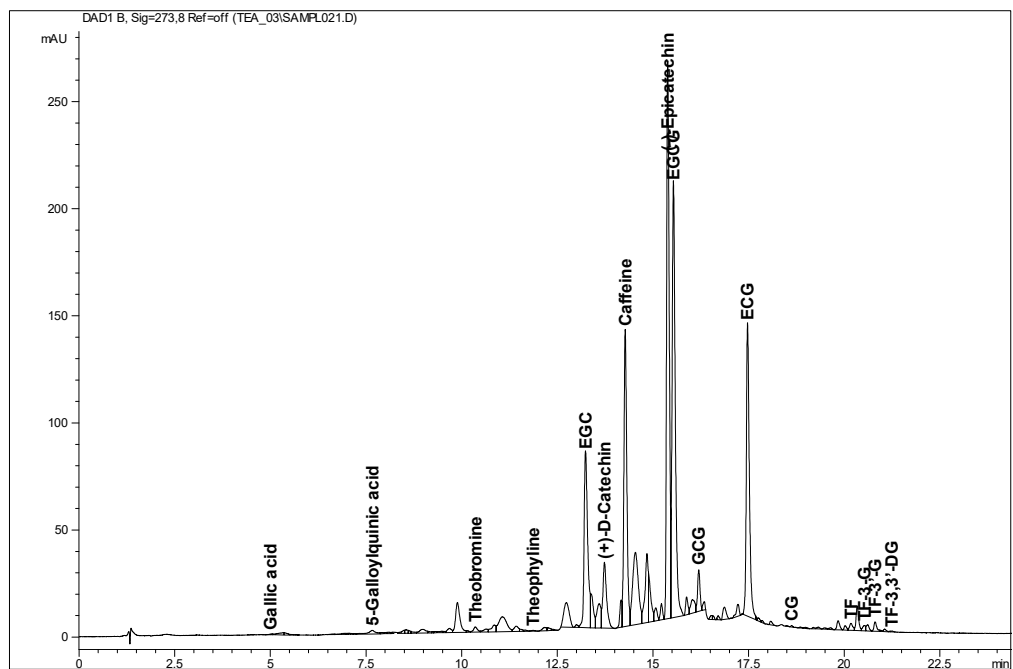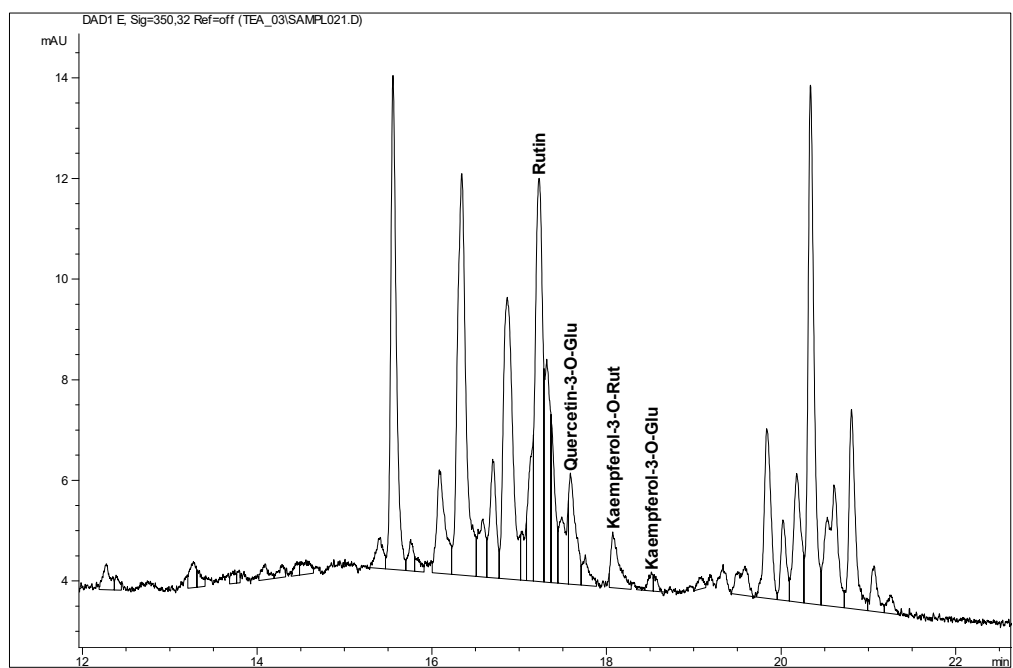

10

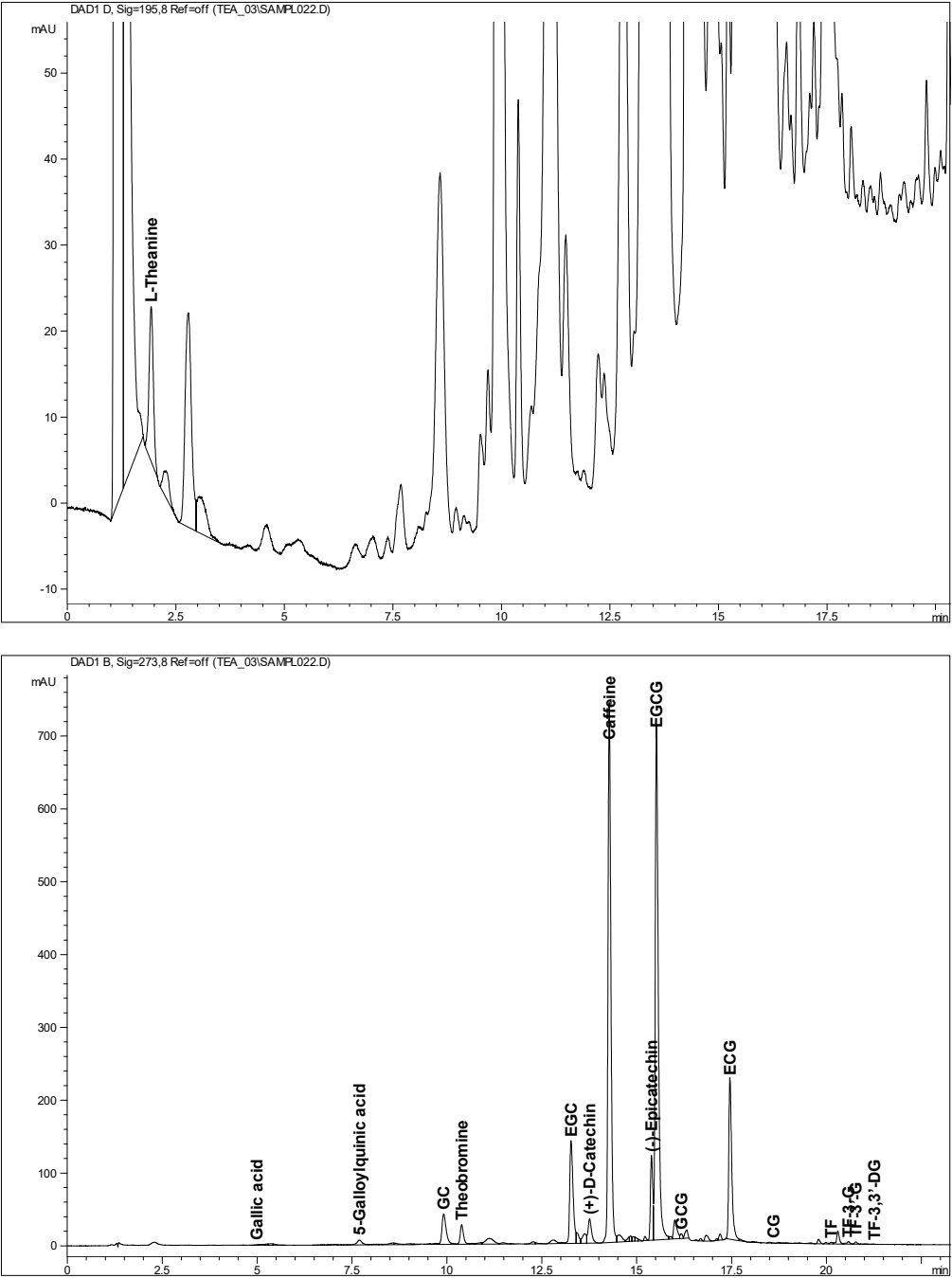

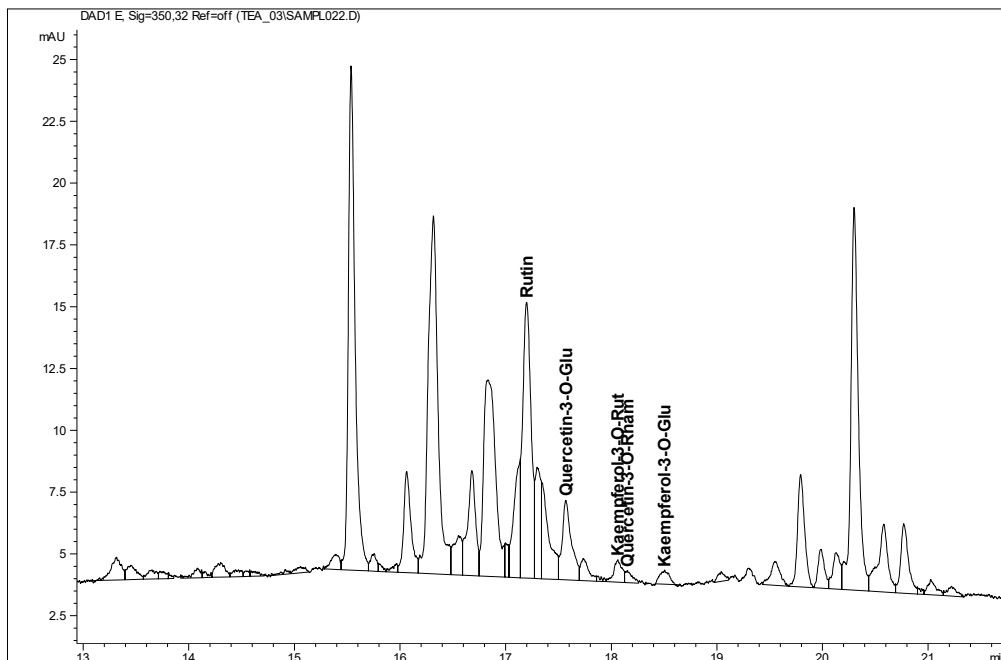

11

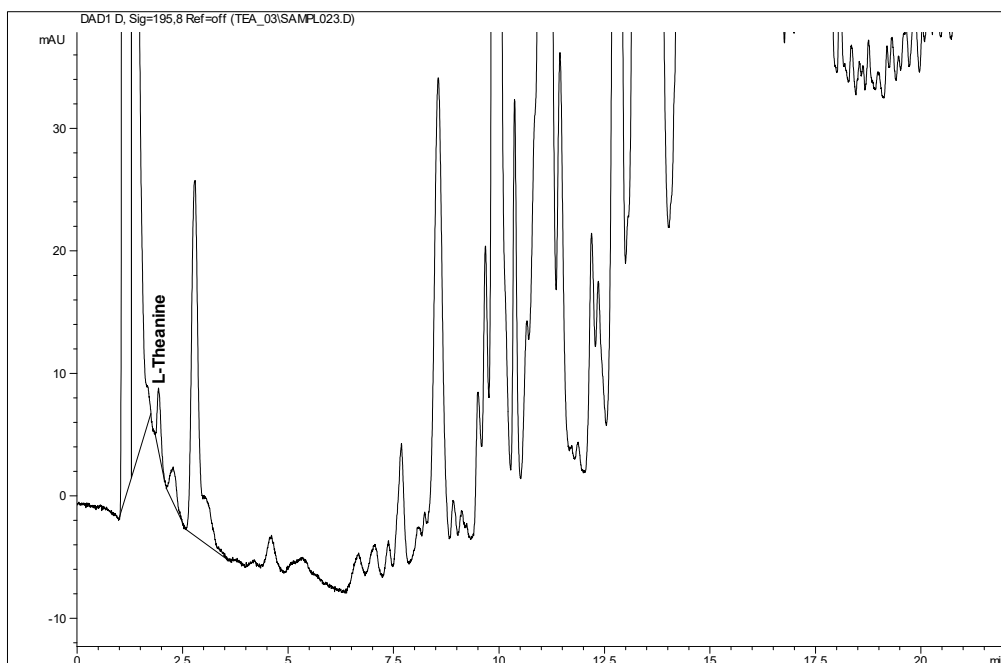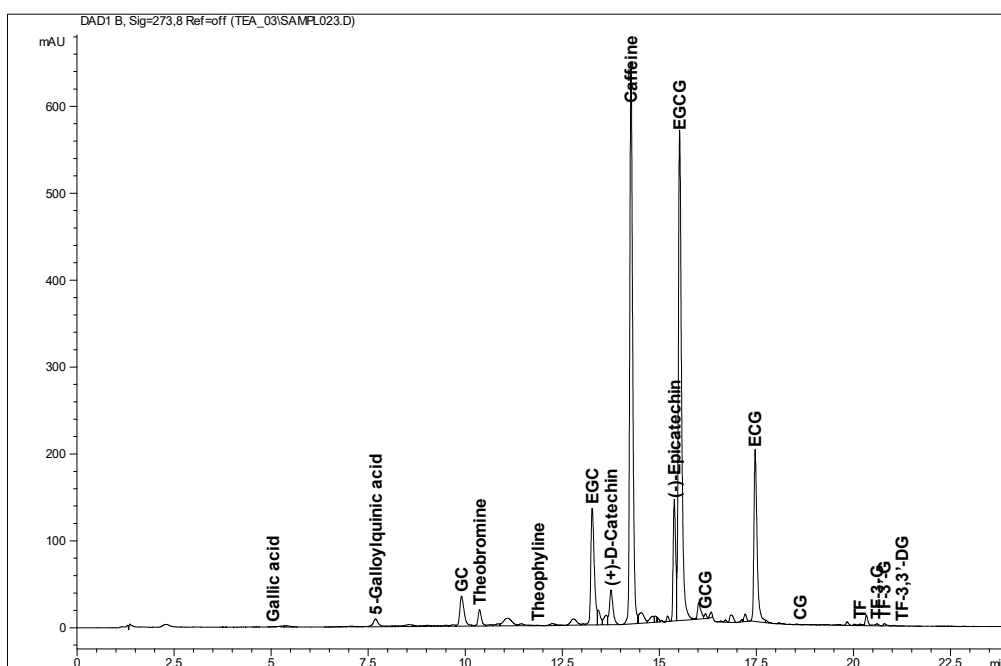

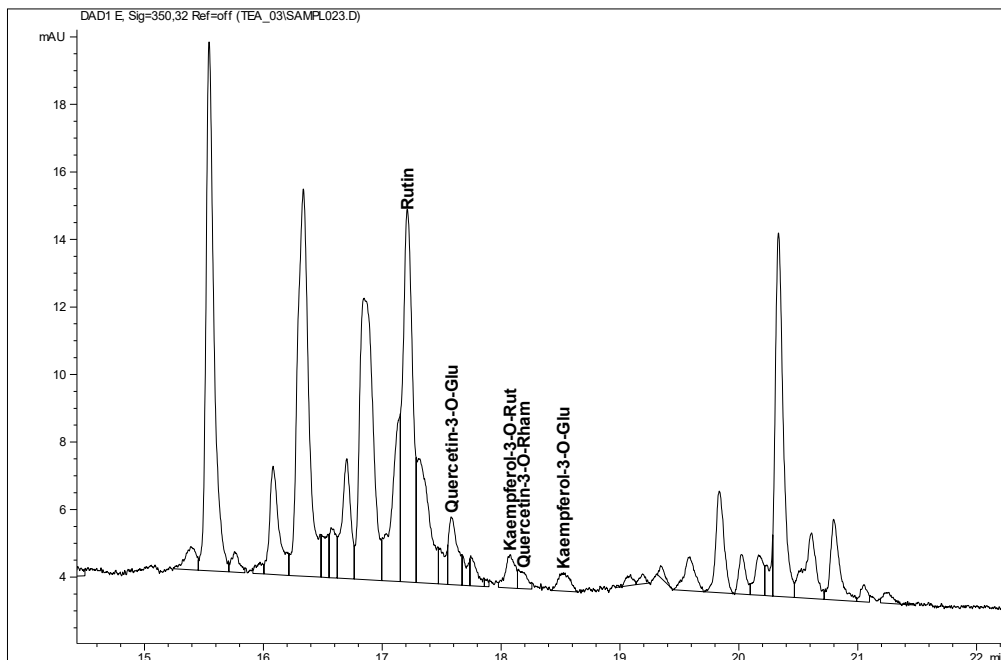

12

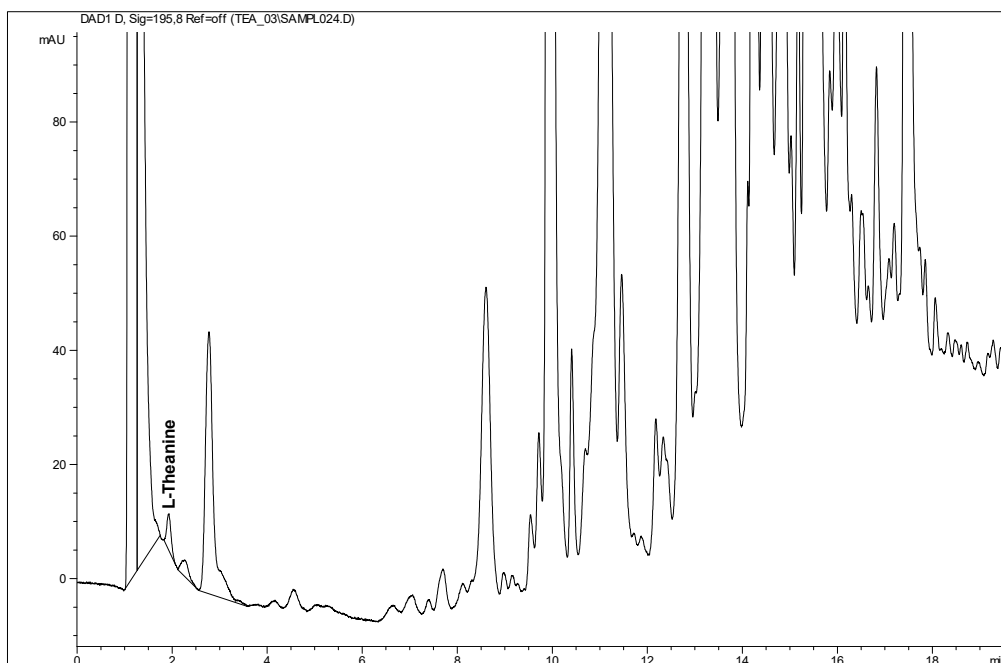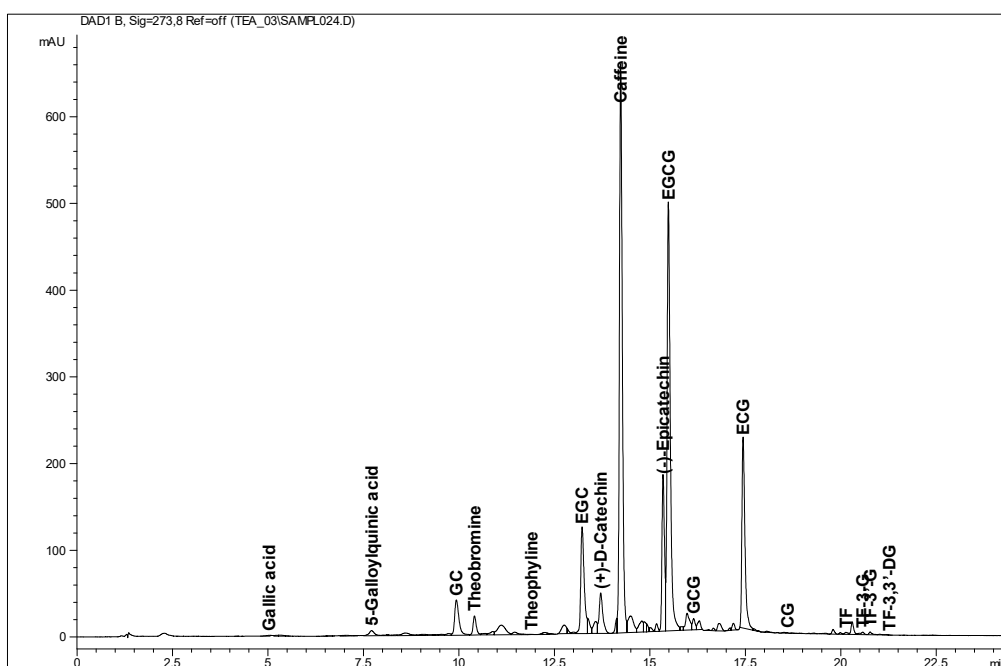

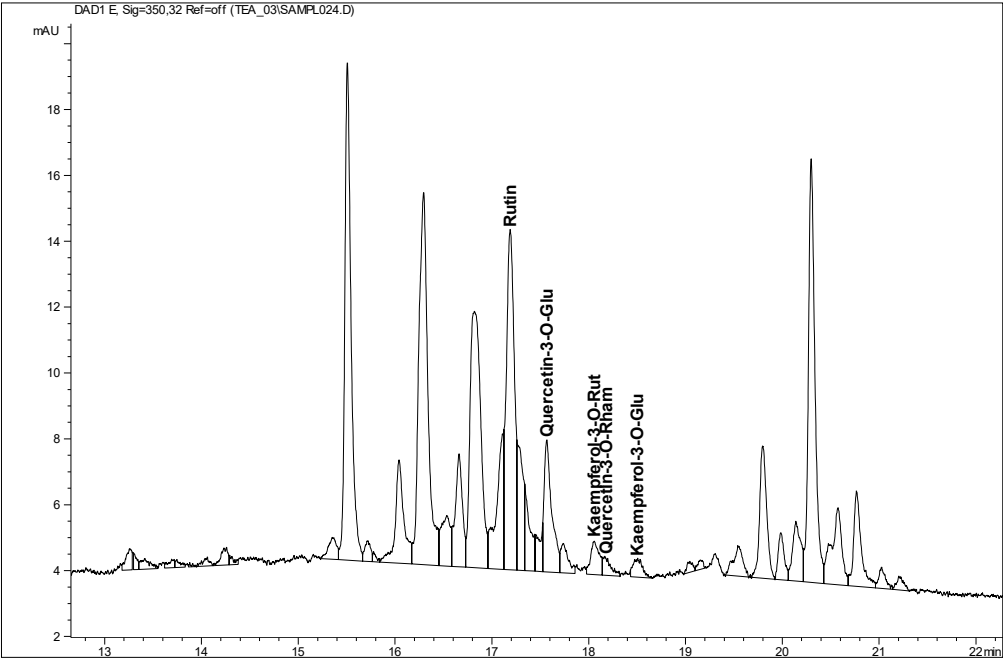

Supplement: Supplementary file 1 [file ijms-25-11726-s001.zip › ijms-3193843-supplementary/Supplementary file 6.pdf]
